# Supplementary material for: Estimating GPS-based social aggregation metrics using collar data
Source: PLoS One. 2026 Apr 9;21(4):e0345546. doi: 10.1371/journal.pone.0345546 (PMC13065019; doi:10.1371/journal.pone.0345546)
Supplement: Attachment — Submitted filename: PONE-D-25-26403_R1_Reviewer comments.pdf [file pone.0345546.s002.pdf]

PLOS ONE

Estimating GPS-based social aggregation metrics using collar data

--Manuscript Draft--

**Manuscript Number:**	PONE-D-25-26403R1
**Article Type:**	Research Article
**Full Title:**	Estimating GPS-based social aggregation metrics using collar data
**Short Title:**	GPS-collar sample size requirements
**Corresponding Author:**	William Janousek<br>US Geological Survey<br>West Glacier, MT UNITED STATES OF AMERICA
**Keywords:**	Adaptive management; animal aggregation; cervids; Chronic wasting disease; telemetry; kernel density estimate; contact rate; sample size
**Abstract:**	Understanding social aggregation patterns in ungulate herds is essential for gaining behavioral insights, optimizing resource use, reducing human-wildlife conflict, and managing disease risk. Chronic wasting disease is the preeminent disease-related threat to cervid populations in North America and knowledge of contact between individuals and spatiotemporal patterns of aggregation provides opportunity to understand and potentially reduce disease risk while supporting sustainable population sizes. Herd density metrics, derived from global positioning system (GPS) data, can be used to inform management decisions. To effectively compare aggregation behavior within and between herds, aggregation metrics must be accurate. However, the consistency of metrics across different GPS collar sample sizes remains unclear and robust studies of big game require understanding how these factors may vary in different contexts. We examined the minimum sample size necessary for reliable calculations of three aggregation metrics: pairwise inter-animal distances, daily proximity rates, and kernel density estimate (KDE) areas. We used GPS collar data from the Jackson and West Green River elk herds (Cervus canadensis) in western Wyoming, USA, that differ in herd size and group structure (single versus multiple sub-groups), representing common practical contexts . Elk locations were acquired for the Jackson herd between 2016 and 2019 and from 2005 to 2010 for the West Green River herd. Herd-specific characteristics substantially influence the sample size necessary for accurate density measurements. As predicted, larger herds with many groups require more GPS collars than small herds with few groups. The sample size needed to accurately estimate aggregation varies by metric, with KDE areas, useful for indexing environmentally transmitted disease risk, generally requiring fewer samples, especially in high-density contexts. The required sample size also varies with seasonal changes in density. During periods of highest density, similar sample sizes are required to estimate inter-animal distances and proximity rates regardless of herd characteristics. Our results have implications for costs associated with studying big game herds, indicating fewer collars may be sufficient in some cases. These insights can aid researchers and managers in determining the appropriate number of GPS collars required for effective herd monitoring and informing relevant aggregation metrics for their management goals.
**Order of Authors:**	William Janousek
	Gavin G. Cotterill
	Olivia J. Lobo
	Eric K. Cole
	Sarah R. Dewey
	Tabitha A. Graves
**Response to Reviewers:**	Thank you for the opportunity to revise our manuscript. We have responded to editor and reviewers' comments line by line in the attached documents. Cheers.
**Additional Information:**	

Powered by Editorial Manager® and ProduXion Manager® from Aries Systems Corporation

Question	Response
**Financial Disclosure**<br><br>Enter a financial disclosure statement that describes the sources of funding for the work included in this submission. Review the submission guidelines for detailed requirements. View published research articles from *PLOS ONE* for specific examples.<br><br>This statement is required for submission and **will appear in the published article** if the submission is accepted. Please make sure it is accurate.<br><br>**Funded studies**<br>Enter a statement with the following details:<br>• Initials of the authors who received each award<br>• Grant numbers awarded to each author<br>• The full name of each funder<br>• URL of each funder website<br>• Did the sponsors or funders play any role in the study design, data collection and analysis, decision to publish, or preparation of the manuscript?<br><br>Did you receive funding for this work?	Yes
Please add funding details.<br>as follow-up to "**Financial Disclosure**<br><br>Enter a financial disclosure statement that describes the sources of funding for the work included in this submission. Review the submission guidelines for detailed requirements. View published research articles from *PLOS ONE* for specific examples.<br><br>This statement is required for submission and **will appear in the published article** if the submission is accepted. Please make sure it is accurate.	Funding was provided by the U.S. Geological Survey Biothreats Program. The funders had no role in study design, data collection and analysis, decision to publish, or preparation of the manuscript.

Powered by Editorial Manager® and ProduXion Manager® from Aries Systems Corporation

**Funded studies**<br>Enter a statement with the following details:<br>• Initials of the authors who received each award<br>• Grant numbers awarded to each author<br>• The full name of each funder<br>• URL of each funder website<br>• Did the sponsors or funders play any role in the study design, data collection and analysis, decision to publish, or preparation of the manuscript?<br><br>Did you receive funding for this work?"	
Please select the country of your main research funder (please select carefully as in some cases this is used in fee calculation).<br>as follow-up to "**Financial Disclosure**<br><br>Enter a financial disclosure statement that describes the sources of funding for the work included in this submission. Review the submission guidelines for detailed requirements. View published research articles from *PLOS ONE* for specific examples.<br><br>This statement is required for submission and **will appear in the published article** if the submission is accepted. Please make sure it is accurate.<br><br>**Funded studies**<br>Enter a statement with the following details:<br>• Initials of the authors who received each award<br>• Grant numbers awarded to each author<br>• The full name of each funder<br>• URL of each funder website<br>• Did the sponsors or funders play any role in the study design, data collection and analysis, decision to publish, or preparation of the manuscript?<br><br>Did you receive funding for this work?"	UNITED STATES - US
**Competing Interests**	The authors have declared that no competing interests exist.

Use the instructions below to enter a competing interest statement for this submission. On behalf of all authors, disclose any competing interests that could be perceived to bias this work—acknowledging all financial support and any other relevant financial or non-financial competing interests.<br><br>This statement is required for submission and **will appear in the published article** if the submission is accepted. Please make sure it is accurate and that any funding sources listed in your Funding Information later in the submission form are also declared in your Financial Disclosure statement.<br><br>View published research articles from *PLOS ONE* for specific examples.<br><br>**NO authors have competing interests**<br><br>Enter: *The authors have declared that no competing interests exist.*<br><br>**Authors with competing interests**<br><br>Enter competing interest details beginning with this statement:<br><br>*I have read the journal's policy and the authors of this manuscript have the following competing interests: [insert competing interests here]*<br><br>* typeset	
**Ethics Statement**<br><br>Enter an ethics statement for this submission. This statement is required if the study involved:<br><br>• Human participants<br>• Human specimens or tissue<br>• Vertebrate animals or cephalopods<br>• Vertebrate embryos or tissues	N/A - all data used in this analysis was previously collected under appropriate permitting and conditions.

Powered by Editorial Manager® and ProduXion Manager® from Aries Systems Corporation

• Field research<br><br>Write "N/A" if the submission does not require an ethics statement.<br><br>General guidance is provided below. Consult the submission guidelines for detailed instructions. **Make sure that all information entered here is included in the Methods section of the manuscript.**	

Powered by Editorial Manager® and ProduXion Manager® from Aries Systems Corporation

**Format for specific study types**<br><br>**Human Subject Research (involving human participants and/or tissue)**<br>• Give the name of the institutional review board or ethics committee that approved the study<br>• Include the approval number and/or a statement indicating approval of this research<br>• Indicate the form of consent obtained (written/oral) or the reason that consent was not obtained (e.g. the data were analyzed anonymously)<br><br>**Animal Research (involving vertebrate animals, embryos or tissues)**<br>• Provide the name of the Institutional Animal Care and Use Committee (IACUC) or other relevant ethics board that reviewed the study protocol, and indicate whether they approved this research or granted a formal waiver of ethical approval<br>• Include an approval number if one was obtained<br>• If the study involved *non-human primates*, add *additional details* about animal welfare and steps taken to ameliorate suffering<br>• If anesthesia, euthanasia, or any kind of animal sacrifice is part of the study, include briefly which substances and/or methods were applied<br><br>**Field Research**<br><br>Include the following details if this study involves the collection of plant, animal, or other materials from a natural setting:<br>• Field permit number<br>• Name of the institution or relevant body that granted permission	
**Data Availability**<br><br>Authors are required to make all data underlying the findings described fully available, without restriction, and from the time of publication. PLOS allows rare exceptions to address legal and ethical concerns. See the PLOS Data Policy and FAQ for detailed information.	No - some restrictions will apply

A Data Availability Statement describing where the data can be found is required at submission. Your answers to this question constitute the Data Availability Statement and **will be published in the article**, if accepted.<br><br>**Important:** Stating 'data available on request from the author' is not sufficient. If your data are only available upon request, select 'No' for the first question and explain your exceptional situation in the text box.<br><br>Do the authors confirm that all data underlying the findings described in their manuscript are fully available without restriction?	
**Describe where the data may be found in full sentences. If you are copying our sample text, replace any instances of XXX with the appropriate details.**<br><br>• If the data are **held or will be held in a public repository**, include URLs, accession numbers or DOIs. If this information will only be available after acceptance, indicate this by ticking the box below. For example: *All XXX files are available from the XXX database (accession number(s) XXX, XXX.*).<br>• If the data are all contained **within the manuscript and/or Supporting Information files**, enter the following: *All relevant data are within the manuscript and its Supporting Information files.*<br>• If neither of these applies but you are able to provide **details of access elsewhere**, with or without limitations, please do so. For example:<br><br>*Data cannot be shared publicly because of [XXX]. Data are available from the XXX Institutional Data Access / Ethics Committee (contact via XXX) for researchers who meet the criteria for access to confidential data.*<br><br>*The data underlying the results presented in the study are available from (include the name of the third party*	Data availability statement reads: West Green River elk herd GPS data are publicly available here: https://doi.org/10.5066/F70K27SF. Jackson elk herd GPS data are available upon request via the National Elk Refuge and Grand Teton National Park (grte_information@nps.gov, attn: Sarah Dewey). Raw aggregation metric data used in this study are available here: https://doi.org/10.5066/P14KSJQM.<br><br>Raw GPS-collar data from elk in the Jackson herd is sensitive due to concerns about potential misuse, particularly related to hunting pressure and poaching. These elk congregate in large numbers within protected landscapes like national parks and wildlife refuges during certain times of the year, making them especially vulnerable if location data is not handled responsibly. Given these factors the collar data is available upon request but continues to be freely shared for research purposes (recent example: Cotterill et al. 2025 Ecosphere).

and contact information or URL).<br>• This text is appropriate if the data are owned by a third party and authors do not have permission to share the data.<br><br>* typeset	
Additional data availability information:	Tick here if your circumstances are not covered by the questions above and you need the journal’s help to make your data available.

Powered by Editorial Manager® and ProduXion Manager® from Aries Systems Corporation

Revised Manuscript - Clean

Click here to access/download;Manuscript;Janousek_GPSAggregationSimula

Estimating GPS-based social aggregation metrics using collar data

William M. Janousek[1*], Gavin G. Cotterill[1], Olivia J. Lobo[1], Eric K. Cole[2], Sarah R. Dewey[3], Tabitha A. Graves[1]

1. U.S. Geological Survey, Northern Rocky Mountain Science Center, West Glacier, MT, United States of America
2. U.S. Fish and Wildlife Service, National Elk Refuge, Jackson, WY, United States of America
3. National Park Service, Grand Teton National Park, Moose, WY, United States of America

[*] Corresponding author

E-mail: wjanousek@usgs.gov (WMJ)

Abstract

Understanding social aggregation patterns in ungulate herds is essential for gaining behavioral insights, optimizing resource use, reducing human-wildlife conflict, and managing disease risk. Chronic wasting disease is the preeminent disease-related threat to cervid populations in North America ~~and~~ knowledge of contact between individuals and spatiotemporal patterns of aggregation provides opportunity to understand and potentially reduce disease risk while supporting sustainable population sizes. Herd density metrics, derived from global positioning system (GPS) data, can be used to inform management decisions. To effectively compare aggregation behavior within and between herds, aggregation metrics must be accurate. However, the consistency of metrics across different GPS collar sample sizes remains unclear

and robust studies of big game require understanding how these factors may vary in different contexts. We examined the minimum sample size necessary for reliable calculations of three aggregation metrics: pairwise inter-animal distances, daily proximity rates, and kernel density estimate (KDE) areas. We used GPS collar data from the Jackson and West Green River elk herds (*Cervus canadensis*) in western Wyoming, USA, that differ in herd size and group structure (single versus multiple sub-groups), representing common practical contexts . Elk locations were acquired for the Jackson herd between 2016 and 2019 and from 2005 to 2010 for the West Green River herd. Herd-specific characteristics substantially influence the sample size necessary for accurate density measurements. As predicted, larger herds with many groups require more GPS collars than small herds with few groups. The sample size needed to accurately estimate aggregation varies by metric, with KDE areas, useful for indexing environmentally transmitted disease risk, generally requiring fewer samples, especially in high-density contexts. The required sample size also varies with seasonal changes in density. During periods of highest density, similar sample sizes are required to estimate inter-animal distances and proximity rates regardless of herd characteristics. Our results have implications for costs associated with studying big game herds, indicating fewer collars may be sufficient in some cases. These insights can aid researchers and managers in determining the appropriate number of GPS collars required for effective herd monitoring and informing relevant aggregation metrics for their management goals.

Keywords: adaptive management, animal aggregation, cervids, chronic wasting disease, telemetry, kernel density estimate, contact rate, sample size

Introduction

Wildlife professionals strive to manage big game herds for optimal social, economic and ecological outcomes [1-2]. These interrelated objectives include increasing wildlife-related recreation opportunities that in turn financially support State management agencies while providing local economies with upwards of $400 billion a year in the United States [3]. They also aim to reduce human-wildlife conflict [4], support ecosystem functioning [5], and control the spread of disease [6]. Increasingly, meeting complex management goals requires more sophisticated information, which must be weighed against increased costs of data collection and analysis. Abundance and herd composition estimates, often generated from visual counts, are commonly used to monitor population trends and set population objectives [7]. These estimates are further enhanced by tying them directly to spatial units, and at ecologically relevant scales, because many biological processes exhibit density-dependence [8-9]. At broad spatial scales, population density estimates help managers meet some of the above objectives. For instance, at the level of agency-defined hunt units, density estimates can directly inform sex- and region-specific hunting quotas or indirectly by identifying priority areas for habitat conservation [10]. In contrast, broad-scale density estimates provide little insight into the interactions, individual movements, and 'local densities' experienced by animals that are most salient to disease control [11]. The proliferation of tracking technology has emerged as a valuable tool to meet the need for detailed monitoring of animal movements and density patterns.

Capturing and marking individual animals for research has a long history in wildlife management [12]. Wildlife tracking via satellite was first demonstrated on elk using radio beacon technology and weather satellites [13]. Contemporary global positioning system (GPS) technology now enables the capture of year-round, high-resolution spatial data which has contributed to its adoption and prevalence in wildlife research [14]. Although methods for

capturing and handling ungulates have improved, these are still expensive operations (e.g., helicopter flights, weeks of staff time) that can induce stress or risk injuring animal subjects [15-16]. More recently, the addition of proximity sensors to collars has emerged as a promising tool capable of recording pairwise interaction rates among individuals, but these too, require direct capture and handling for deployment [17]. As a result, GPS collars are only ever deployed on a subset of individuals to inform processes representative of the larger population which can create challenges for contact analyses [18]. Nevertheless, GPS collar datasets are ubiquitous and permit the analysis of movement paths, conspecific contact rates, and spatiotemporal density patterns that inform disease transmission risk [19-20], and in some cases, are retroactively used in epidemiological investigations [21].

As more animals that occupy shared space and have the potential to interact are tracked, GPS data reveal some of the conditions that facilitate transmission of infectious diseases [22-24], including the timing, location, and structure of herd aggregations. Previous works established links between herd aggregations and brucellosis [25-26] and bovine tuberculosis transmission [27]. Chronic wasting disease (CWD), a fatal prion disease spreading rapidly through cervid populations in North America and Fennoscandia [28-29], exhibits characteristics of density dependent transmission [30]. Location data can also be used to predict how a disease would spread if introduced to a novel population by assessing the spatiotemporal patterns of transmission risk [31]. Furthermore, management strategies to reduce aggregation and decrease the potential for disease transmission are being directly informed by GPS location data [32-35]. Even so, the predictions made by any study can only be used effectively if aggregation metrics produced by GPS collar data are accurate.

The topic of sample size requirements has been persistent in conversations about the use of GPS collar data for population-level inference for ecological processes including survival, resource selection, and home range estimation (reviewed by [14]). However, sample size requirements to accurately estimate aggregation patterns of big game herds has received less attention (but refer to [36-38] for studies on other taxa). Multi-agency working groups have identified the management of ungulate density, either through adaptive management protocols or experimental means, as integral to addresssing the spread of CWD transmission in North America [39]. Further, targeted reductions in host density through hunting can help manage CWD prevalence [40] and are projected to improve achievement of long-term population objectives [41-42]. As a result, managers and researchers may be interested in devoting some of their resources towards addressing emerging disease threats by better understanding patterns of aggregation in ungulate populations. Informing the knowledge gap of sample size requirements can help direct limited conservation resources including funds and personnel hours.

We focus on three metrics used to understand animal aggregation with implications for direct and indirect pathways of disease transmission: pairwise inter-animal distances, daily proximity rates (a measure of time spent at close distances), and kernel density estimate (KDE) areas [43]. All three metrics are easily calculated using traditional GPS datasets [44] and are loosely analogous to related concepts in social network analysis wherein they describe individual-level, intermediate-level, and group-level measures, respectively [45]. Mathematical disease models traditionally treated transmission processes as frequency- or density-dependent [46], although empirical studies often estimate modes of transmission that fall somewhere between the two extremes [11, 47]. Inter-animal distances, proximity rates, and KDE area estimates also follow a frequency-density continuum and thus can inform disease risk. Inter-

animal distances (combined with some threshold to index contact probability) align conceptually with contact frequencies, KDEs with densities, and proximity rates are intermediary. Outside disease research, collar-based distance metrics have also been used for analyzing animal social behaviors [38, 48] and can form the basis for building formal interaction networks [49].

We investigated the minimum number of GPS collars needed to accurately estimate aggregation metrics using GPS collar data from two elk herds (*Cervus canadensis*) of varying size and structure at two times of the year. Relative ungulate herd densities and contact probabilities fluctuate seasonally (e.g., summer versus winter) with changing biotic and abiotic conditions [33]. During winter, elk herds concentrate on relatively small winter ranges where individuals interact with more conspecifics (homogenous mixing; Fig 1a). During summer, elk disperse across the landscape which reduces relative densities, and results in fewer conspecific contacts (heterogeneous mixing; Figs 1b-c). We predicted that estimation accuracy of the aggregation metrics would vary by season and reflect different degrees of connectivity among individuals and groups of individuals within a herd. Accurate aggregation metrics should be obtainable at lower sample sizes during times of higher density regardless of absolute herd size. In contrast, during low-density conditions, a large population defined by multiple distinct sub-groups with heterogenous mixing should require a larger minimum sample size than a small herd that aggregates as a single semi-homogenous group. We compare the three metrics and discuss how managers can leverage existing and planned GPS datasets to help manage disease risk for their ungulate populations.

Fig 1. Network Structure Concepts. Diagram showing predictions of variation in network structure within wildlife populations of co-occurring individuals. The full network structure is unobserved without known locations of all individuals but with an adequate sample of the

population metrics of aggregation can identify the degree of connectivity. Network structure can range from a single highly mixed homogenous group (a) to heterogeneous with multiple unconnected groups (c). As network structure changes different numbers of sampled individuals may be necessary for accurate evaluation.

Materials and methods

We investigated aggregation metrics for two elk herds in western Wyoming, USA. Jackson elk (JKSN) were collared on the National Elk Refuge (NER, 43.4805 N, -110.7428 W) and West Green River elk (WGR) were collared on Fossil Butte National Monument (FOBU, 41.8558 N, -110.7615 W). The JKSN herd inhabit the Middle Rockies ecoregion defined by steep mountains dominated by coniferous forest and shrub- and grass-covered foothills (1,500–3,000 m elevation [50]). During this study, collared elk from the JKSN herd occupied an 1,822 km^2 area encompassing their wintering grounds on NER and summer range in the Teton and Gros Ventre Ranges, including portions of Grand Teton and Yellowstone National Parks to the west and north and Bridger Teton National Forest to the east (Fig 2, 3a-b). WGR elk inhabit the foothill shrublands and low conifer-dotted mountains of the Wyoming Basin ecoregion (1,500–2,200 m elevation [50]). Elk from the WGR herd occupied a 730 km^2 area, including their winter range on Fossil Butte National Monument and summer range in the surrounding Tunp Range which mainly consists of Bureau of Land Management lands (Fig 2, 3c-d). Both herds experience predation risk from mountain lions (*Puma concolor*), coyotes (*Canis latrans*), black bears (*Ursus americanus*), grizzly bears (*Ursus arctos*), and gray wolves (*Canis lupus*), with the latter two being more prevalent in the JKSN herd range [51].

Fig 2. Study Area Map. Map showing the study region and locations of the National Elk Refuge and Fossil Butte National Monument in the state of Wyoming, home to the wintering grounds of the Jackson elk herd (JKSN) and West Green River elk herd (WGR), respectively. The background relief is based on the SRTM 30-m digital elevation model provided by NASA JPL [52]. Inset map indicating location of Wyoming derived from U.S. Census Bureau boundary data [53].

The JKSN herd has a population of ~11,000 elk [54] and is comprised of disaggregated subgroups (Fig 3a, heterogeneous mixing) during months of lowest density and a homogenous group during high density winter months (Fig 3b, [55-56]). The WGR herd is estimated to number ~600 elk based on winter herd counts and maintains a consistent homogenous to semi-homogenous group structure (one to few groups) throughout the year (Fig 3c-d, [55-56]). We evaluated GPS collar data with 1.5-hour fix rates for 68 female elk collared between 2016 and 2019 from the JKSN herd after excluding five elk with successful daily fix rates below 0.95 [33]. The WGR herd was monitored with GPS collars recording locations at 5-hour intervals from 2005 to 2010. During that period, 61 female elk were collared for varying lengths of time [55]. Due to declining fix rates over time in the WGR herd, we calculated the rolling seven-day average fix rate for each collar and when the average fell below 0.75, we excluded all subsequent days from our analysis.

Fig 3. Seasonal Variation in Herd Structure. Map showing 90% contour from kernel density estimates for elk from the Jackson (JKSN, purple) and West Green River (WGR, blue) herds during low (a,c) and high density (b,d) periods in Wyoming, USA. The location of the National

Elk Refuge and Fossil Butte National Monument are indicated by black bordered polygons. The background relief is based on the SRTM 30-m digital elevation model provided by NASA JPL [52].

We calculated pairwise inter-animal distances, daily proximity rates (a measure of the proportion of the day that two animals are within 500 m of each other), and kernel density estimate areas (KDEs) over the full period available for each herd [33]. In previous work [33], we explored other distance thresholds (100 and 250 m) as alternatives to a 500-m threshold for daily proximity rates and found comparable relationships across methods. At 500 m, we observed the greatest daily fluctuations in proximity rates, which tracked daily changes in abiotic conditions [33]. This indicates that the 500-m threshold is more useful for monitoring proximity rates at fine temporal scales within the context of this study system. Evaluating multiple distances can inform choice of appropriate distance thresholds and may be unique to each study, taxa, spatiotemporal scale, and method or frequency of data collection.

To assess sample size needs, we selected months of lowest and highest densities based on the aggregation metrics. The low- and high-density months included July 2017 (N = 45 elk) and March 2019 (N = 32 elk), respectively for the JKSN herd, and April 2006 (N= 23 elk) and October 2006 (N= 19 elk) for the WGR herd. Because inter-animal distances and proximity rates are daily metrics, we selected the 15th day of each month for our comparison. We summarized pairwise inter-animal distances using the first quartile distance, because this summary is most sensitive to changes in aggregation [43], and we summarized proximity rates using the pairwise mean. We calculated herd-level KDEs and corresponding areas for 50%, 70%, and 90% contours at monthly timescales. We used the default reference bandwidth smoothing parameterization in our KDE estimation, based on the standard deviation of spatial coordinates and sample size [57],

to broadly capture the area used by each collared elk population [58]. The smoothing bandwidth can greatly influence how KDEs are drawn, and practitioners can consider their specific ecological context, project goals, and data structure when calculating KDEs. Our interest centered on the effects of changing sample sizes on metric accuracy rather than fine tuning KDEs for more explicit ecological investigation.

For each herd-month-metric combination, we randomly sampled collared elk, incrementing between two and the maximum number of collared elk and resampled 1,000 times with replacement per increment. Assuming the full dataset represented the most accurate value (hereafter baseline value), we assessed variation in aggregation metrics across subsets of collared elk by calculating the percent difference of metrics between the subset and the maximum sample size to determine the accuracy of metrics as sample size increased. For each sample size, we calculated the proportion of subsets within 20% of the baseline value of each aggregation metric. We considered the accuracy threshold to be met when ≥90% of simulations at a given sample size fell within 20% of the baseline value. All aggregation metrics were calculated using the *wildagg* package for program R [44, 59].

Results

The larger JKSN herd typically required larger sample sizes than the smaller WGR herd. Periods when herds were dispersed, with only semi-homogenous and heterogenous mixing (lowest density) required larger minimum sample sizes to achieve accurate measures of all aggregation metrics than periods of homogenous mixing (highest density), regardless of herd identity, (Table 1, Fig 4). During low density months, minimum required sample sizes ranged from 1.9–6.7 times larger than in months of high density for the WGR herd and 1.5–4.2 times larger for the JKSN herd, depending on the aggregation metric (Table 1, Figs 4e-h). To

accurately estimate all metrics for the WGR herd, minimum sample sizes of 22 and 16 GPS-collared elk were required under low- and high-density conditions, respectively, which amounts to 96% and 84% of the maximum available sample size for this herd (Table 1). To accurately estimate all metrics simultaneously for the JKSN herd, sample sizes of 42 and 24 GPS-collared elk were required under low- and high-density conditions, respectively, which reflect 93% and 75% of the maximum available sample size for this herd (Table 1).

Table 1. Minimum sample size required for aggregation metrics

	WGR		JKSN	
Metric	Low Density	High Density	Low Density	High Density
90% KDE Area	13	3	37	11
70% KDE Area	18	3	36	14
50% KDE Area	20	3	36	24
1st Quartile Inter-Animal Distance	21	16	28	14
Proximity rate	21	11	42	10

Minimum sample size required to ensure aggregation metrics are within 20% of the baseline value calculated at maximum available sample size for the Jackson (JKSN) and West Green River (WGR) elk herds in Wyoming, USA during periods of low and high density.

Fig 4. Sample Size Accumulation Curves for Each Aggregation Metric. The proportion of simulations at each sample size that are within 20% of the baseline value calculated at maximum available sample size for elk from the Jackson and West Green River herds in Wyoming, USA. Each aggregation metric is calculated during high- and low-density periods for each herd (a-d, e-h, respectively). Aggregation metrics include the daily 1st quartile inter-elk distance (a,e), mean daily proximity rates (b,f), and the area of 90% (c,g) and 50% contours from kernel density estimates (d,h). Dashed lines indicate when the proportion of simulations within 20% of the

baseline value is ≥ 90% (numerical values shown in Table 1). Accumulation curves are fitted using loess smoothing methods for visualization.

Fewer collared elk were needed to produce accurate estimates for KDEs compared to other aggregation metrics for the smaller WGR herd regardless of density conditions (N=3 under high-density and as few as N = 11 under low-density conditions). In contrast, aggregation metrics requiring the most and fewest collared elk to reach the accuracy threshold were more variable for the JKSN herd. Under high density months, 50% KDE area calculations required the most samples and proximity rates required the fewest samples whereas proximity rates and inter-animal distance metrics required the most and fewest samples during periods of low density for the JKSN herd.

As minimum sample size increases under periods of high density for both elk herds, the proportion of simulations within 20% of the baseline value approach 100% well before reaching the maximum available sample size (Figs 4a-d; but see inter-animal distance estimates for the WGR herd in Fig 4a). In contrast, under low density months, the proportion of simulation estimates within 20% of the baseline value of inter-animal distances, proximity rates, and 50% KDE contour area reach 90% within three sample size increments below the maximum available sample size for the WGR herd (Figs 4e-f and 4h). Proximity rate estimates at low density for the JKSN herd was the only metric to not asymptote at higher sample sizes (Fig 4f).

Discussion

Herd size, seasonal density patterns and resulting network complexity modulate the minimum sample size required to accurately estimate aggregation metrics for GPS-collared elk. When elk are widely dispersed during the summer months, the overlap of space use between

individuals decreases, requiring a larger sample to capture aggregation patterns for the herd. In contrast, when herds are tightly grouped, as they are on wintering grounds, an individual's space use commonly overlaps with others in the herd. The metrics robust to small sample sizes varied both within and between herds, indicating that herd context is an important consideration when determining the optimum sample size and density metrics for studying aggregation behavior in big game populations.

Our results have considerable implications for how managers assess disease transmission risk in ungulate herds. Most pressing in western big game populations is the issue of CWD, which has multiple transmission pathways, adding value to consideration of multiple metrics. Chronic wasting disease can be transmitted by direct contact between individuals or indirectly through the contaminated environment (reviewed by [30]). Assessing risk of direct transmission requires approaches like proximity rates (referred to as contact rates in disease ecology), whereas assessing risk of indirect transmission requires estimating overall space use and defining spatial areas of high concentrations which can be achieved through KDE contours. Our analyses indicate proximity rate calculations need more samples due to this metric requiring GPS-collared animals to be within some minimum distance threshold where interactions occur. For demonstration purposes we used a distance threshold of 500 m in this analysis, which provided robust estimates for understanding the drivers of elk aggregation in previous work [33], but smaller distance thresholds may be required to capture disease-relevant interactions [60]. As distance thresholds decrease, the number of collars in a population will likely need to increase. In contrast, the small sample sizes needed to accurately calculate KDEs likely result from the pooling of location information across individuals to define space use of the herd as a collective.

Transmission of any disease is not uniform across space, time, or from individual to

individual [61]. Comparisons such as the one we conducted between herds with different sizes and structure can increase our understanding of how transmission risk may differ across unique populations. Our findings that reasonable accuracy for KDE area, proximity rates and inter-animal distance calculations became obtainable for two distinct herds during two periods of different densities and social structure confirm that GPS collar data can provide accurate aggregation metrics to inform decision-making. Studies predict that herd density reduction prior to CWD establishment can result in better long-term outcomes (e.g., higher elk abundance) than actions taken after CWD is established in herds [41-42]. This lends support to multi-agency objectives based on adaptive management approaches targeting ungulate density in various ways to address disease spread [39].

In our study the highest densities and aggregations of elk occur during the winter, when the sexes are together in space and time. In other systems or taxa, the composition of collared individuals (either by sex, age-class, or other characteristics) will need to align with knowledge about transmission dynamics unique to each disease. The mode of transmission (indirect versus direct) will necessarily dictate which aggregations metrics (e.g., KDE for indirect) may be most informative. Researchers should also consider the density characteristics of the population when determining how many collars to deploy and potentially conduct similar resampling analyses as this study presents to fully understand the implications of sampling decisions. This may be especially true for multi-year studies when the opportunity to adjust collar deployments exists. Although our results indicate some aggregation metrics can be accurately measured at low sample sizes, ensuring a representative sample of a herd is integral to avoid sampling individuals that differ considerably in their spatiotemporal movement patterns thereby inducing biased

aggregation estimates. Finally, controlling for collar performance as we have done by censoring individuals with low fix rates, can minimize potentially spurious estimates of aggregation.

The aggregation metrics calculated in this study were evaluated within the paradigm of interacting, mobile animals. Fixed points on the landscape, for example attractants like anthropogenic food sources or mineral licks, can also serve as aggregation hotspots increasing the likelihood of direct contact between individuals and between individuals and a pathogen contaminated environment [30, 62]. Using the R package *wildagg* [44], as we did to estimate aggregation metrics, managers could treat the locations of attractants as additional 'individuals' to quantify interactions between their wildlife populations and potential point sources of increased pathogen deposition. When the locations of attractants are unknown, cluster analyses may help identify aggregation hotspots [63], which can be described by the aggregation metrics explored in this study.

Managers of big game herds rely on aggregation data to make decisions that can have broad economic and ecological implications such as setting tag quotas, limiting disease transmission risk, or identifying areas with potential for human-wildlife conflict. These results provide researchers and managers with insight into how many GPS collars should be deployed depending on the size and behavior of their herds. With this knowledge, researchers and managers can direct conservation dollars for greatest return on investment.

Acknowledgements

We thank the Grand Teton Association, U.S. Fish and Wildlife Service, Fossil Butte National Monument, BLM Kemmerer Field Office, Wyoming Game and Fish Department, and the Bridger-Teton National Forest for supporting GPS collar deployment on the National Elk

Refuge and Fossil Butte National Monument. Any use of trade, firm or product names is for descriptive purposes only and does not imply endorsement by the U.S. Government.

Data availability statement

West Green River elk herd GPS data are publicly available here: https://doi.org/10.5066/F70K27SF [64]. Jackson elk herd GPS data are available upon request via the National Elk Refuge and Grand Teton National Park (grte_information@nps.gov, attn: Sarah Dewey). Raw aggregation metric data used in this study are available here: https://doi.org/10.5066/P14KSJQM [65].

References

1. Gordon IJ, Hester AJ, Festa-Bianchet MA. The management of wild large herbivores to meet economic, conservation and environmental objectives. Journal of Applied Ecology. 2004 Dec;41(6):1021-31.
2. Robinson KF, Fuller AK, Hurst JE, Swift BL, Kirsch A, Farquhar J, et al. Structured decision making as a framework for large-scale wildlife harvest management decisions. Ecosphere. 2016 Dec;7(12):e01613.
3. U.S. Department of the Interior [U.S. DOI], U.S. Fish and Wildlife Service. National Survey of Fishing, Hunting, and Wildlife-Associated Recreation. 2022 [Cited 2025 Apr 10]. Available from: https://www.fws.gov/media/2022-national-survey-fishing-hunting-and-wildlife-associated-recreation.

4. Jones JD, Proffitt KM, Paterson JT, Almberg ES, Cunningham JA, Loveless KM. Elk responses to management hunting and hazing. The Journal of Wildlife Management. 2021 Nov;85(8):1721-38.
5. Côté SD, Rooney TP, Tremblay JP, Dussault C, Waller DM. Ecological impacts of deer overabundance. Annu. Rev. Ecol. Evol. Syst.. 2004 Dec 15;35(1):113-47.
6. Lambert S, Thébault A, Rossi S, Marchand P, Petit E, Toïgo C, et al. Targeted strategies for the management of wildlife diseases: the case of brucellosis in Alpine ibex. Veterinary Research. 2021 Sep 14;52(1):116.
7. Rabe MJ, Rosenstock SS, deVos Jr JC. Review of big-game survey methods used by wildlife agencies of the western United States. Wildlife Society Bulletin. 2002 Apr 1:46-52.
8. Gaillard JM, Festa-Bianchet M, Yoccoz NG. Population dynamics of large herbivores: variable recruitment with constant adult survival. Trends in ecology & evolution. 1998 Feb 1;13(2):58-63.
9. Storm DJ, Samuel MD, Rolley RE, Shelton P, Keuler NS, Richards BJ, et al. Deer density and disease prevalence influence transmission of chronic wasting disease in white-tailed deer. Ecosphere. 2013 Jan;4(1):1-4.
10. Krausman PR, Bleich VC. Conservation and management of ungulates in North America. International Journal of Environmental Studies. 2013 Jun 1;70(3):372-82.
11. Ferrari MJ, Perkins SE, Pomeroy LW, Bjørnstad ON. Pathogens, social networks, and the paradox of transmission scaling. Interdisciplinary perspectives on infectious diseases. 2011;2011(1):267049.

12. Whitney K. History of wildlife tracking technologies. In Oxford Research Encyclopedia of Environmental Science 2022 Mar 23. https://doi.org/10.1093/acrefore/9780199389414.013.725.

13. Craighead FC, Craighead JJ, Cote CE, Buechner HK. Satellite and ground radiotracking of elk. 1972. Available from: https://ntrs.nasa.gov/citations/19720017418.

14. Hebblewhite M, Haydon DT. Distinguishing technology from biology: a critical review of the use of GPS telemetry data in ecology. Philosophical Transactions of the Royal Society B: Biological Sciences. 2010 Jul 27;365(1550):2303-12.

15. Katzner TE, Arlettaz R. Evaluating contributions of recent tracking-based animal movement ecology to conservation management. Frontiers in Ecology and Evolution. 2020 Jan 24;7:519.

16. Lahoz-Monfort JJ, Magrath MJ. A comprehensive overview of technologies for species and habitat monitoring and conservation. BioScience. 2021 Oct;71(10):1038-62.

17. Tosa MI, Schauber EM, Nielsen CK. Localized removal affects white-tailed deer space use and contacts. The Journal of Wildlife Management. 2017 Jan;81(1):26-37.

18. Cross PC, Creech TG, Ebinger MR, Heisey DM, Irvine KM, Creel S. Wildlife contact analysis: emerging methods, questions, and challenges. Behavioral Ecology and Sociobiology. 2012 Oct;66:1437-47.

19. Cross PC, Lloyd-Smith JO, Bowers JA, Hay CT, Hofmeyr M, Getz WM. Integrating association data and disease dynamics in a social ungulate: bovine tuberculosis in African buffalo in the Kruger National Park. InAnnales Zoologici Fennici 2004 Jan 1 (pp. 879-892). Finnish Zoological and Botanical Publishing Board.

20. Wilber MQ, Yang A, Boughton R, Manlove KR, Miller RS, Pepin KM, et al. A model for leveraging animal movement to understand spatio-temporal disease dynamics. Ecology Letters. 2022 May;25(5):1290-304.

21. Malmberg JL, Alder J, Killion H, Buttke D, Pepin KM, Wittemyer G. Cross–Species Transmission at the Wildlife–Livestock Interface: A case study of epidemiological inference from mule deer GPS collar data. Ecology and Evolution. 2025 Apr;15(4):e71182.

22. Schauber EM, Storm DJ, Nielsen CK. Effects of joint space use and group membership on contact rates among white-tailed deer. The Journal of wildlife management. 2007 Feb;71(1):155-63.

23. Williams DM, Dechen Quinn AC, Porter WF. Informing disease models with temporal and spatial contact structure among GPS-collared individuals in wild populations. PLoS One. 2014 Jan 7;9(1):e84368.

24. Sargeant GA, Wild MA, Schroeder GM, Powers JG, Galloway NL. Spatial network clustering reveals elk population structure and local variation in prevalence of chronic wasting disease. Ecosphere. 2021 Dec;12(12):e03781.

25. Cross PC, Cole EK, Dobson AP, Edwards WH, Hamlin KL, Luikart G, et al. Probable causes of increasing brucellosis in free-ranging elk of the Greater Yellowstone Ecosystem. Ecological Applications. 2010 Jan;20(1):278-88.

26. Proffitt KM, Anderson N, Lukacs P, Riordan MM, Gude JA, Shamhart J. Effects of elk density on elk aggregation patterns and exposure to brucellosis. The Journal of wildlife management. 2015 Apr;79(3):373-83.

27. Wilber MQ, Pepin KM, Campa III H, Hygnstrom SE, Lavelle MJ, Xifara T, et al. Modelling multi-species and multi-mode contact networks: implications for persistence of bovine tuberculosis at the wildlife–livestock interface. Journal of Applied Ecology. 2019 Jun;56(6):1471-81.

28. Richards B. Distribution of chronic wasting disease in North America. United States Geological Survey, National Wildlife Health Center. 2025 [Cited 2025 Mar 4]. Available from: https://www.usgs.gov/media/images/distribution-chronic-wasting-disease-north-america-0.

29. Tranulis MA, Gavier-Widén D, Våge J, Nöremark M, Korpenfelt SL, Hautaniemi M, et al. Chronic wasting disease in Europe: new strains on the horizon. Acta Veterinaria Scandinavica. 2021 Dec;63:1-5.

30. Escobar LE, Pritzkow S, Winter SN, Grear DA, Kirchgessner MS, Dominguez-Villegas E, et al. The ecology of chronic wasting disease in wildlife. Biological Reviews. 2020 Apr;95(2):393-408.

31. Wilson-Henjum G, Ricci L, Stoner DC, Longshore K, Manlove KR. Desert bighorn sheep home range and disease transmission risk responses to temporally dynamic environmental variation. The Journal of Wildlife Management. 2025 Apr;89(3):e22715.

32. Pepin KM, Davis AJ, Beasley J, Boughton R, Campbell T, Cooper SM, et al. Contact heterogeneities in feral swine: implications for disease management and future research. Ecosphere. 2016 Mar;7(3):e01230.

33. Janousek WM, Graves TA, Berman EE, Chong GW, Cole EK, Dewey SR, et al. Human activities and weather drive contact rates of wintering elk. Journal of Applied Ecology. 2021 Mar;58(3):667-76.

34. Jennelle CS, Walter WD, Crawford J, Rosenberry CS, Wallingford BD. Movement of white-tailed deer in contrasting landscapes influences management of chronic wasting disease. The Journal of Wildlife Management. 2022 Nov;86(8):e22306.

35. Janousek WM, Cole EK, Dewey SR, Graves TA. Informing adaptive management to reduce ungulate aggregations: A case study involving winter feeding of elk. Wildlife Society Bulletin. 2025 Mar;49(1):e1574.

36. Sequeira AM, Heupel MR, Lea MA, Eguíluz VM, Duarte CM, Meekan MG, et al. The importance of sample size in marine megafauna tagging studies. Ecological Applications. 2019 Sep;29(6):e01947.

37. Shimada T, Thums M, Hamann M, Limpus CJ, Hays GC, FitzSimmons NN, et al. Optimising sample sizes for animal distribution analysis using tracking data. Methods in Ecology and Evolution. 2021 Feb;12(2):288-97.

38. He P, Klarevas-Irby JA, Papageorgiou D, Christensen C, Strauss ED, Farine DR. A guide to sampling design for GPS-based studies of animal societies. Methods in Ecology and Evolution. 2023 Aug;14(8):1887-905.

39. Western Association of Fish and Wildlife Agencies [WAFWA]. Recommendations for adaptive management of chronic wasting disease in the West. WAFWA Wildlife Health Committee and Mule Deer Working Group. Edmonton, Alberta, Canada and Fort Collins, Colorado, USA. 2017. Available from: https://wafwa.org/wpdm-

package/recommendations-for-adaptive-management-of-chronic-wasting-disease-in-the-west/.

40. Moss WE, Binfet J, Hall LE, Allen SE, Edwards WH, Jennings-Gaines JE, et al. The effectiveness of harvest for limiting wildlife disease: Insights from 20 years of chronic wasting disease in Wyoming. Ecological Applications. 2025 Jan;35(1):e3089.

41. Cook JD, Cross PC. Decision analysis in support of the National Elk Refuge bison and elk management plan: U.S. Geological Survey Scientific Investigations Report 2024–5119; 2025. 5 chap. (A–E), variously paged, https://doi.org/10.3133/sir20245119.

42. Cross PC, Cook JD, Cole, EK. Predictions of elk and chronic wasting disease dynamics at the National Elk Refuge in Jackson, Wyoming, and surrounding areas, chap. B of Cook, JD, and Cross PC, eds., Decision analysis in support of the National Elk Refuge bison and elk management plan: U.S. Geological Survey Scientific Investigations Report 2024–5119; 2025. 22 p., https://doi.org/10.3133/sir20245119B.

43. Graves TA, Yarnall MJ, Johnston AN, Preston TM, Chong GW, Cole EK, et al. Eyes on the herd: Quantifying ungulate density from satellite, unmanned aerial systems, and GPS collar data. Ecological Applications. 2022 Jul;32(5):e2600.

44. Janousek WM, Graves TA. wildagg: Wildlife Aggregation Estimation & Visualization. U.S. Geological Survey software release. Reston, Virginia. Version 0.1.2. 2021. Available from: https://doi.org/10.5066/P9DTQEJS.

45. Wey T, Blumstein DT, Shen W, Jordán F. Social network analysis of animal behaviour: a promising tool for the study of sociality. Animal behaviour. 2008 Feb 1;75(2):333-44.

46. Anderson RM, May RM. Population biology of infectious diseases: Part I. Nature. 1979 Aug 2;280(5721):361-7.

47. Smith MJ, Telfer S, Kallio ER, Burthe S, Cook AR, Lambin X, et al. Host–pathogen time series data in wildlife support a transmission function between density and frequency dependence. Proceedings of the National Academy of Sciences. 2009 May 12;106(19):7905-9.

48. Benson JF, Keiter DA, Mahoney PJ, Allen BL, Allen L, Álvares F, et al. Intrinsic and environmental drivers of pairwise cohesion in wild Canis social groups. Ecology. 2025 Jan;106(1):e4492.

49. Kaur P, Ciuti S, Ossi F, Cagnacci F, Morellet N, Loison A, et al. A protocol for assessing bias and robustness of social network metrics using GPS based radio-telemetry data. Movement Ecology. 2024 Aug 6;12(1):55.

50. Chapman SS, Bryce SA, Omernik JM, Despain DG, ZumBerge J, Conrad M. Ecoregions of Wyoming (color poster with map, descriptive text, summary tables, and photographs): Reston, Virginia, U.S. Geological Survey (map scale 1:1,400,000). 2004. Available from: https://www.epa.gov/eco-research/ecoregion-download-files-state-region-8#pane-48.

51. Buskirk SW, Wild Mammals of Wyoming and Yellowstone National Park. 2016 Univ of California Press.

52. Farr TG, Rosen PA, Caro E, Crippen R, Duren R, Hensley S, et al. The shuttle radar topography mission. Reviews of geophysics. 2007 Jun;45(2).

53. U.S. Census Bureau. Cartographic Boundary File: Current State and Equivalent Entities "cb_2018_us_state_500k". U.S. Department of Commerce. 2018. https://www.census.gov/geographies/mapping-files/time-series/geo/carto-boundary-file.html.

54. U.S. Fish and Wildlife Service [USFWS]. Bison and Elk Management Step-down Plan. National Elk Refuge, Grand Teton National Park, Wyoming. Lakewood, CO: U.S. Department of the Interior, U.S. Fish and Wildlife Service. National Park Service. 2019. Available from: https://iris.fws.gov/APPS/ServCat/Reference/Profile/154124.

55. Mikle NL, Graves TA, Olexa EM. To forage or flee: lessons from an elk migration near a protected area. Ecosphere. 2019 Apr;10(4):e02693.

56. Altmann M. Social behavior of elk, *Cervus canadensis nelsoni*, in the Jackson Hole area of Wyoming. Behaviour. 1952 Jan 1:116-43.

57. Worton BJ. Kernel methods for estimating the utilization distribution in home-range studies. Ecology. 1989 Feb;70(1):164-8

58. Gitzen RA, Millspaugh JJ, Kernohan BJ. Bandwidth selection for fixed-kernel analysis of animal utilization distributions. The Journal of Wildlife Management. 2006 Dec;70(5):1334-44.

59. R Core Team. R: A language and environment for statistical computing. R Foundation for Statistical Computing, Vienna, Austria. 2021[cited 2025 Apr 5].

60. Habib TJ, Merrill EH, Pybus MJ, Coltman DW. Modelling landscape effects on density–contact rate relationships of deer in eastern Alberta: implications for chronic wasting disease. Ecological Modelling. 2011 Aug 10;222(15):2722-32.

61. Albery GF, Sweeny AR, Becker DJ, Bansal S. Fine-scale spatial patterns of wildlife disease are common and understudied. Functional Ecology. 2022 Jan;36(1):214-25.

62. Plummer IH, Johnson CJ, Chesney AR, Pedersen JA, Samuel MD. Mineral licks as environmental reservoirs of chronic wasting disease prions. PloS one. 2018 May 2;13(5):e0196745.

63. Cotterill GG, Cole EK, Cross PC, Dewey SR, Wise BL, Graves TA. Elk personality and anthropogenic food subsidy: Managing conflict and migration loss. Ecosphere. 2025 Aug;16(8):e70344.

64. Graves TA, Mikle NL, Olexa EM. West Green River elk herd locations in southwestern Wyoming, 2005-2010; 2019 [cited 2025 Apr 5]. U.S. Geological Survey data release. Available from: https://doi.org/10.5066/F70K27SF.

65. Janousek WM, Cotterill, GG, Lobo, O, Cole, EK, Dewey, SR, Graves, TA. Estimates of Elk Aggregation Metrics Calculated Across Varying Sample Sizes from Two Herds in Western Wyoming, USA. 2025 [cited 2025 July 23]. US Geological Survey data release https://doi.org/10.5066/P14KSJQM.

Figure 1

Click here to access/download;Figure;Fig1.tif

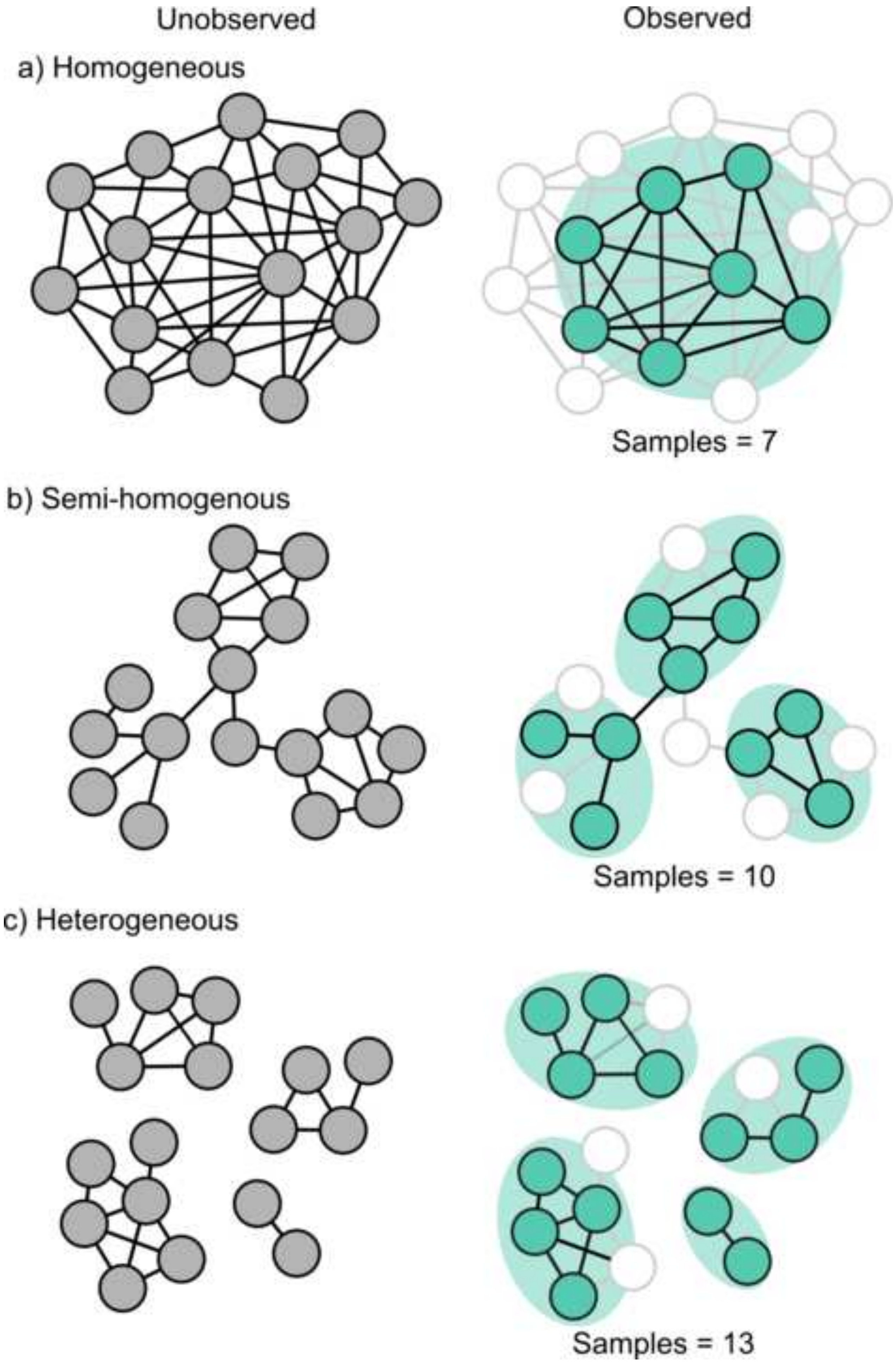

Figure 2

Click here to access/download;Figure;Fig2.tif

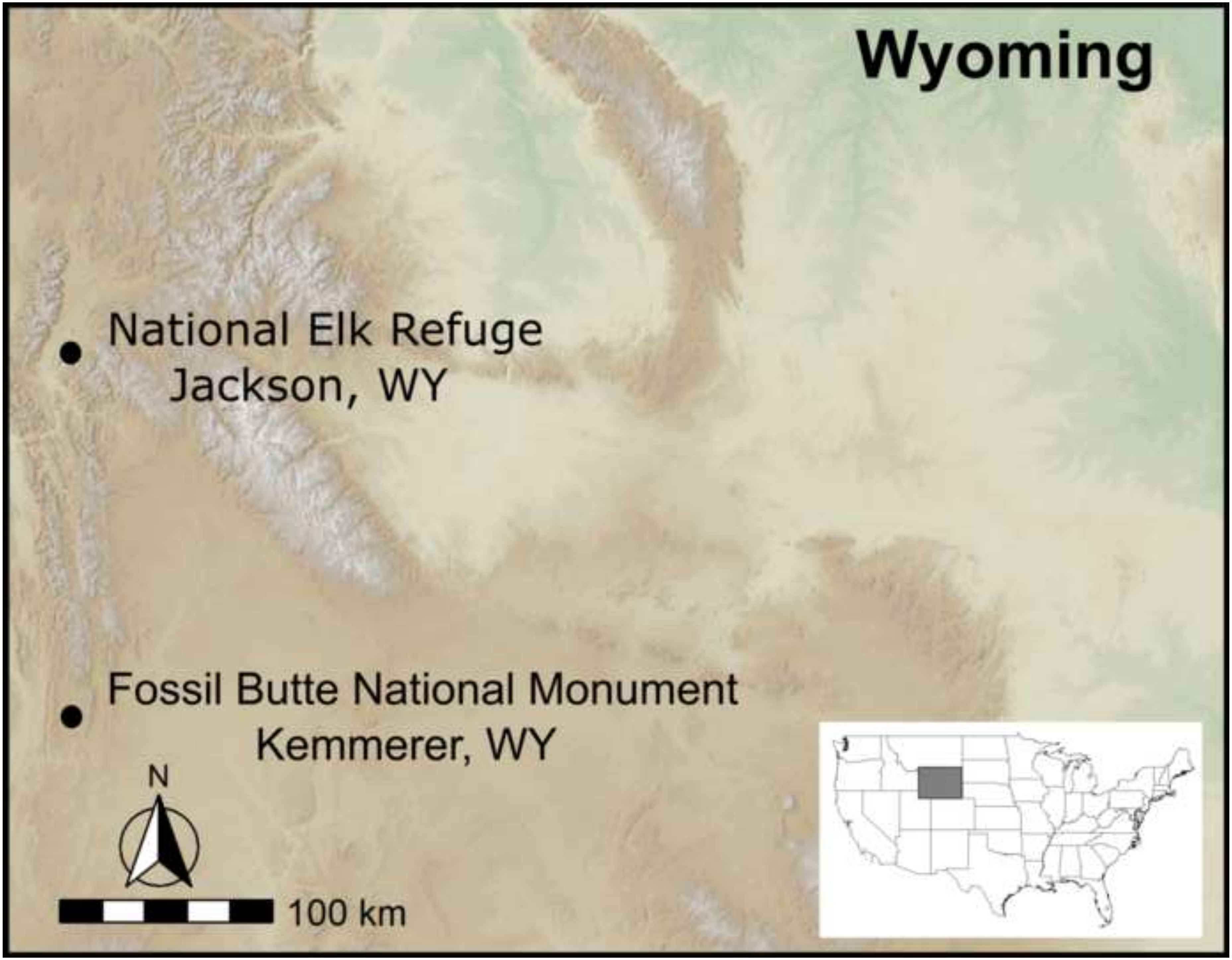

Figure 3 Click here to access/download;Figure;Fig3.tif

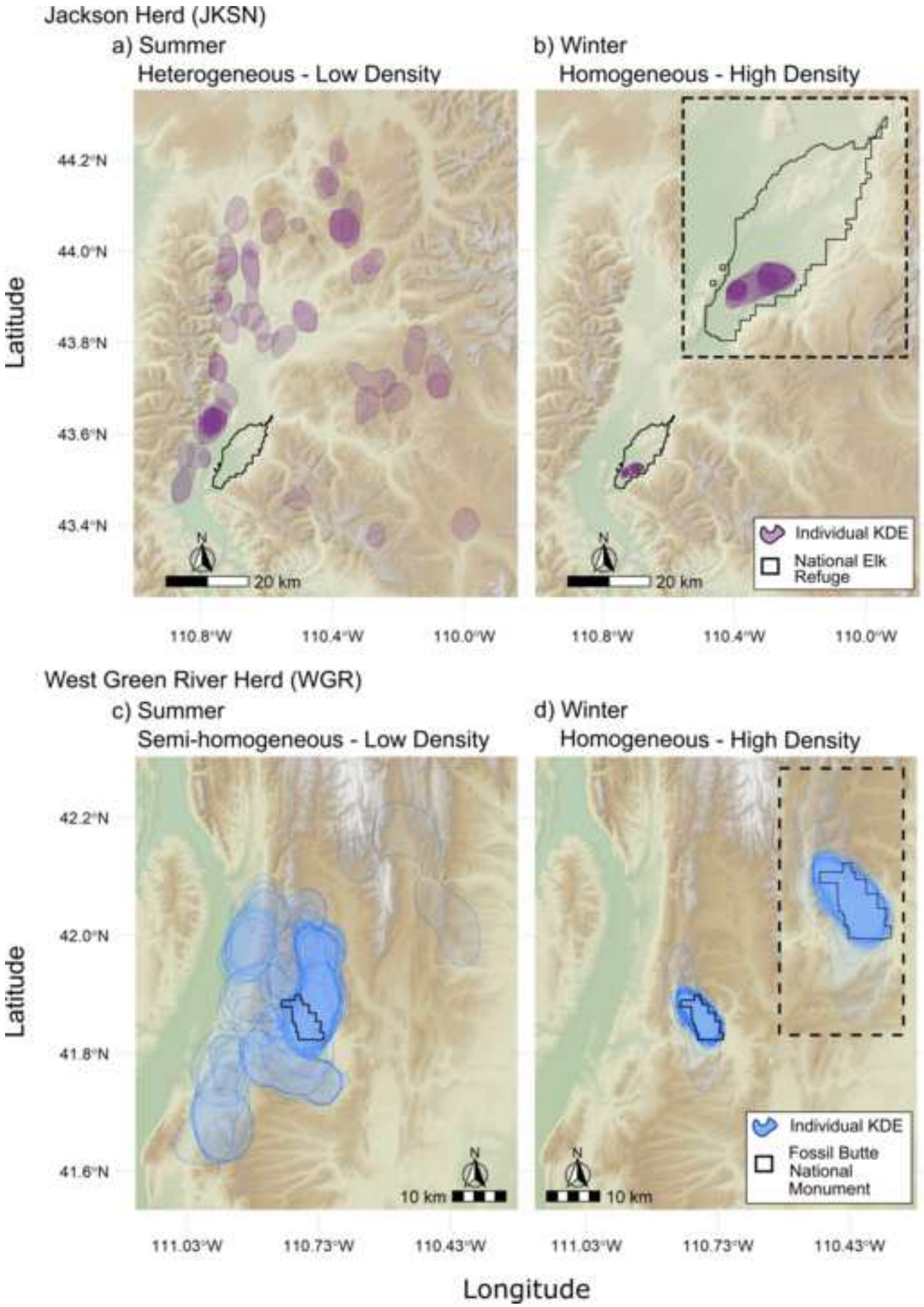

Figure 4

Click here to access/download;Figure;Fig4.tif

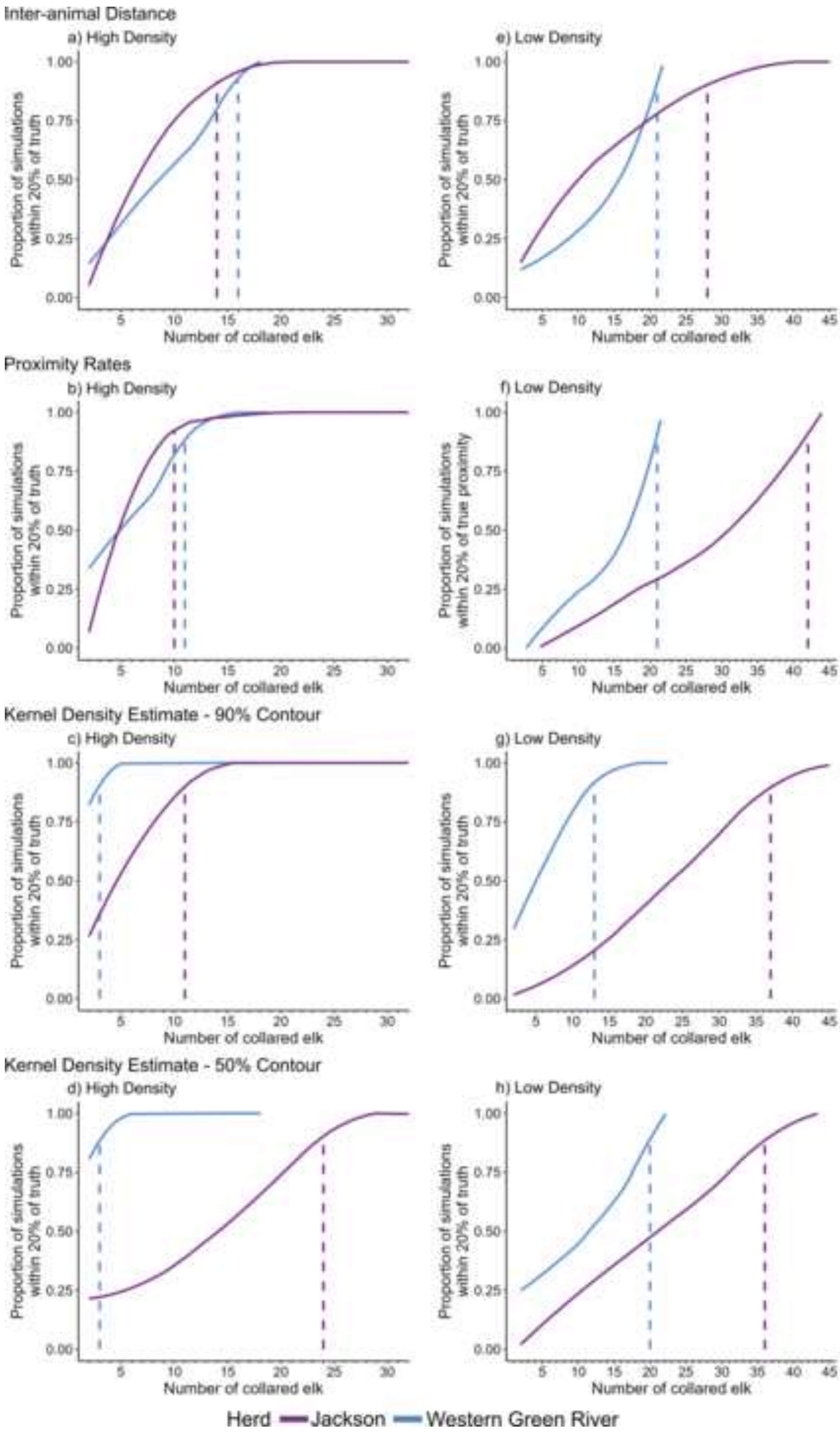

This draft manuscript is distributed solely for purposes of scientific peer review. Its content is deliberative and predecisional, so it must not be disclosed or released by reviewers. Because the manuscript has not yet been approved for publication by the U.S. Geological Survey (USGS), it does not represent any official USGS finding or policy.

Estimating GPS-based social aggregation metrics using collar data

William M. Janousek[1*], Gavin G. Cotterill[1], Olivia Lobo[1], Eric K. Cole[2], Sarah R. Dewey[3], Tabitha A. Graves[1]

1. U.S. Geological Survey, Northern Rocky Mountain Science Center, West Glacier, MT, United States of America
2. U.S. Fish and Wildlife Service, National Elk Refuge, Jackson, WY, United States of America
3. National Park Service, Grand Teton National Park, Moose, WY, United States of America

[*] Corresponding author

E-mail: wjanousek@usgs.gov (WMJ)

Abstract

Understanding social aggregation patterns in ungulate herds is essential for gaining behaviouoral insights, optimizing resource use, reducing human-wildlife conflict, and managing disease risk. Ungulate herd density metrics, derived from global positioning system (GPS) data, play a crucial role in informing management decisions. Chronic wasting disease is the

preeminent disease-related threat to cervid populations ~~of big game~~ in North America and knowledge of contact between individuals and spatiotemporal patterns of aggregation~~s~~ provides opportunity to understand and potentially reduce disease risk~~,~~ ~~lessening decreases in~~while supporting sustainable population sizes. ~~Ungulate h~~Herd density metrics, derived from global positioning system (GPS) data, play a crucial role in informing management decisions. To effectively compare aggregation behavior within and between herds, aggregation metrics must be accurate. However, the consistency of metrics across different GPS collar sample sizes remains unclear and robust studies of big game require understanding how these factors may vary in different contexts. We examined the minimum sample size necessary for reliable calculations of three aggregation metrics: pairwise inter-animal distances, daily proximity rates, and kernel density estimate (KDE) areas. We used GPS collar data from the ~~two~~ Jackson and West Green River elk herds (*Cervus canadensis*) in western Wyoming, USA, that differ in herd size and group~~ing~~ structure (single ~~group~~ versus multiple sub-groups), representing common practical contexts ~~managers encounter in practice~~. Elk locations ~~data~~ were~~as~~ acquired for the Jackson herd between 2016 and 2019 and from 2005 to 2010 for the West Green River herd. Herd-specific characteristics substantially influence the sample size necessary for accurate density measurements. As predicted, larger herds with many groups require more GPS collars than small herds with few groups. The sample size needed to accurately estimate aggregation varies by metric, with KDE~~kernel density estimate~~ areas, useful for indexing environmentally-transmitted disease risk, generally requiring fewer samples, especially in high-density contexts. ~~However,~~ ~~t~~The required sample size also varies with seasonal changes in density.~~,~~ D~~and d~~uring periods of highest density, similar sample sizes are required ~~for some metrics~~to estimate inter-animal distances and proximity rates regardless of herd ~~size~~characteristics. ~~The sample size needed to~~

~~accurately estimate aggregation also varies by metric with kernel density estimate areas generally requiring fewer samples of a population especially in high density contexts.~~ Our results have implications for costs associated with studying big game herds, ~~showing~~ indicating fewer collars may be sufficient in some cases. These insights will aid researchers and managers in determining the appropriate number of GPS collars required for effective herd monitoring and inform the most relevant aggregation metrics for their management goals.

Keywords: adaptive management, animal aggregation, cervids, chronic wasting disease, telemetry, kernel density estimate, contact rate, sample size

Introduction

Wildlife professionals strive to manage big game herds for optimal social, economic and ecological outcomes [1-2]. These interrelated objectives include increasing wildlife-related recreation opportunities that in turn financially support State management agencies while providing local economies with upwards of $400B a year in the United States [3]. They also aim to reduce human-wildlife conflict [4], support ecosystem functioning [5], and control the spread of disease [6]. Increasingly, meeting complex management goals requires more sophisticated information, which must be weighed against increased costs of data collection and analysis. Abundance and herd composition estimates, often generated from visual counts, are commonly used to monitor population trends and set population objectives [7]. These estimates are further enhanced by tying them directly to spatial units, and at ecologically-relevant scales, because many biological processes exhibit density-dependence [8-9]. At broad spatial scales, population density estimates help managers meet some of the above objectives. For instance, at the level of agency-defined hunt units, ~~these~~ density estimates can ~~help by~~ directly ~~regulating~~ inform sex-

and region-specific hunting quotas or indirectly by identifying priority areas for habitat conservation [10]. In contrast, broad-scale density estimates provide little insight into the interactions, individual movements, and ‘local densities’ experienced by animals that are most salient to disease control [11]. The proliferation of tracking technology has emerged as a valuable tool to meet the need for detailed monitoring of animal movements and density patterns.~~fills this need.~~

Capturing and marking individual animals for research ~~purposes~~ has a long history in wildlife management [12]. Wildlife tracking via satellite was~~,~~ first demonstrated on elk using radio beacon technology and weather satellites [13].~~, and later~~ Contemporary global positioning system (GPS) technology now enables the capture~~ing~~ of year-round, high-resolution spatial data which has contributed to ~~their~~ its adoption and prevalence in wildlife research [14]. While methods for capturing and handling ungulates have improved, these are still expensive~~costly~~ operations (e.g., helicopter flights, weeks of staff time) that can induce stress or risk ~~harming~~ injuring animal subjects [15-16]. More recently, the addition of proximity sensors to collars ~~-~~has emerged as a promising tool capable of recording pairwise ~~contacts~~ interaction rates ~~between~~ among individuals~~ have emerged as a promising tool~~, but these too, require direct capture and handling for deployment [17]. As a result, GPS collars are only ever deployed on a subset of individuals to inform processes representative of the larger population which can create challenges for contact analyses [18]. Nevertheless, GPS collar datasets are ubiquitous and permit the analysis of movement paths, conspecific contact rates, and spatiotemporal density patterns that inform ~~that provide important information regarding~~ disease transmission risk [19-20], and in some cases, are retroactively used in ~~inform~~ epidemiological investigations [21].

~~When multiple animals are collared on shared ranges~~As more animals that occupy shared space and have the potential to interact are tracked, GPS data reveal some of the conditions that facilitate transmission of infectious diseases [22-24], including the timing, location, and structure of herd aggregations. Previous works established links between herd aggregations and brucellosis [25-26] and bovine tuberculosis transmission [27]. Chronic wasting disease (CWD), a fatal prion disease spreading rapidly through cervid populations in North America and Fennoscandia [28-29],~~ also~~ exhibits characteristics of density dependent transmission~~ce~~ [30]. Location data can also be used to predict how a disease would spread if introduced to a novel population by assessing the spatiotemporal patterns of transmission risk [31]. Furthermore, management strategies to reduce aggregation and decrease the potential for disease transmission are being directly informed by GPS location data [32-35]. Even so, the predictions made by any study can only be used effectively if aggregation metrics produced by GPS collar data are accurate.

The topic of sample size requirements has been persistent in conversations about the use of GPS collar data for population-level inference for ecological processes including survival, resource selection, and home range estimation (reviewed by [14]). However, sample size requirements to accurately estimate aggregation patterns of big game herds has received less attention (but refer to [36-38] for studies on other taxa). Multi-agency working groups have identified management of ungulate density either through adaptive management protocols or experimental means as approaches to address the spread of CWD transmission in North America [39]. Further, targeted reductions in host density through hunting can help manage CWD prevalence [40] and are projected to improve achievement of long-term population objectives [41-42]. As a result, managers and researchers may be interested in devoting some of their

resources towards addressing emerging disease threats ~~via~~ by better understanding patterns of aggregation in ungulate populations. Informing the knowledge gap of sample size requirements will help direct limited conservation resources including funds and personnel hours.

We focus on three metrics used to understand animal aggregation with implications for direct and indirect pathways of disease transmission: pairwise inter-animal distances, daily proximity rates (a measure of time spent at close distances), and kernel density estimate (KDE) areas [43]. All three metrics are easily calculated using traditional GPS datasets [44] and are loosely analogous to related concepts in social network analysis wherein they describe individual-level, intermediate-level, and group-level measures, respectively [45]. Mathematical disease models traditionally treated transmission processes as frequency- or density-dependent [46], although empirical studies often estimate modes of transmission that fall somewhere between the two extremes [11, 47]. Inter-animal distances, proximity rates, and KDE area estimates also follow a frequency-density continuum and thus can inform disease risk. Inter-animal distances (combined with some threshold to index contact probability) align conceptually with contact frequencies, KDEs with densities, while proximity rates are intermediary. Outside disease research, collar-based distance metrics have ~~a long history of use~~also been used for analyzing animal social behaviors [38, 48] and can form the basis for building formal interaction networks [49].

We investigated the minimum number of GPS collars needed to accurately estimate aggregation metrics using GPS collar data from two elk herds (*Cervus canadensis*) of varying size and structure at two times of the year. Relative ungulate herd densities and contact probabilities fluctuate seasonally (e.g., summer versus winter) with changing biotic and abiotic conditions [33]. During winter, elk herds concentrate on relatively small winter ranges where

individuals interact with more conspecifics (homogenous mixing; Fig 1a). During summer, elk disperse across the landscape which reduces relative densities, and results in fewer conspecific contacts (heterogeneous mixing; Figs 1b-c). We predicted that estimation accuracy of the three aggregation metrics would vary by season~~the number of GPS collars needed to accurately estimate the aggregation of big game herds varies depending on timing of aggregation assessments~~ which reflect different degrees of connectivity among individuals and groups of individuals within a herd. Accurate aggregation metrics should be obtainable at lower sample sizes during times of higher density regardless of absolute herd size. In contrast, during low-density conditions, a large population defined by multiple distinct sub-groups with heterogenous mixing should require a larger minimum sample size than a small herd that aggregates as a single semi-homogenous group. We compare the three metrics and discuss how managers can leverage existing and planned GPS datasets to help manage disease risk for their ungulate populations.

Fig 1. Network Structure Concepts. Diagram showing predictions of variation in network structure within wildlife populations of co-occurring individuals. The full network structure is unobserved without known locations of all individuals but with an adequate sample of the population metrics of aggregation can identify the degree of connectivity. Network structure can range from a single highly mixed homogenous group (a) to heterogeneous with multiple unconnected groups (c). ~~Populations with less connectivity~~As network structure changes different numbers of sampled individuals may be necessary ~~require more samples~~ for accurate evaluation.

Materials and methods

We investigated aggregation metrics for two elk herds in western Wyoming, USA. ~~Data from the~~ Jackson elk ~~herd~~ (JKSN) ~~comes from elk~~were collared on the National Elk Refuge

(NER, 43.4805 N, -110.7428 W) and ~~data from the~~ West Green River elk ~~herd~~ (WGR) ~~comes~~ were ~~from elk~~ collared on Fossil Butte National Monument (FOBU, 41.8558 N, -110.7615 W). The JKSN herd ~~is found in~~inhabit the Middle Rockies ecoregion defined by steep mountains dominated by coniferous forest and shrub- and grass-covered foothills (1,500–3,000m elevation [50]). During this study, collared elk from the JKSN herd ~~were located observed within~~occupied an 1,822 km^2 area encompassing their wintering grounds on NER and summer range in the Teton and Gros Ventre Ranges, including portions of Grand Teton and Yellowstone National Parks to the west and north and Bridger Teton National Forest to the east (Fig 2, 3a-b~~a~~). ~~The~~ WGR elk ~~herd~~ inhabit ~~occurs within~~ the foothill shrublands and low conifer-dotted mountains of the Wyoming Basin ecoregion (1,500–2,200m elevation [50]). Elk from the WGR herd occupied~~were found observed within~~ a 730 km^2 area, including their winter range on Fossil Butte National Monument and summer range in the surrounding Tunp Range which mainly consists of Bureau of Land Management lands (Fig 2, 3c-d~~b~~). B~~While b~~oth herds experience predation risk from mountain lions (*Puma concolor*), coyotes (*Canis latrans*), black bears (*Ursus americanus*),~~the JKSN herd also live within the range of~~ grizzly bears (*Ursus arctos*) and gray wolves (*Canis lupus*), with the latter two being more prevalent in the JKSN herd range [51].

Fig 2. Study Area Map. Map ~~showing 90% contour from kernel density estimates for elk from the Jackson (JKSN, purple) and West Green River (WGR, blue) herds during low (a,c) and high density (b,d) periods in Wyoming, USA. The~~ showing the study region and locations of the National Elk Refuge and Fossil Butte National Monument ~~are indicated by black bordered polygons~~in the state of Wyoming, home to the wintering grounds of the Jackson elk herd (JKSN)

Formatted: Font: Italic

Formatted: Font: Italic

Commented [WJ1]: New reference numbering begin here

Formatted: Highlight

and West Green River elk herd (WGR), respectively. The background relief is based on the SRTM 30m digital elevation model provided by NASA JPL [52~~1~~].

The JKSN herd ~~maintain~~has a population of ~11,000 elk [53~~2~~], ~~and during months of lowest density~~ and is comprised of ~~many~~ disaggregated subgroups (Fig 3a, heterogeneous mixing) during months of lowest density and a homogenous group during high density winter months ~~,~~(Fig 3b, [54-55]). ~~while the~~The WGR herd is ~~smaller~~ estimated to number ~~(~~ ~600 elk ~~according to~~ based on winter herd counts, ~~in FOBU)~~ and maintains a consistent homogenous to semi-homogenous group structure (one to few groups) throughout the year (Fig ~~1~~3c-d, [~~53~~ 54-55]). We evaluated GPS collar data with 1.5-hour fix rates for 68 female elk collared between 2016 and 2019 from the JKSN herd after excluding five elk with successful daily fix rates below 0.95 [33]. The WGR herd was monitored with GPS collars recording locations at 5-hour intervals from 2005 to 2010. During that period, 61 female elk were collared for varying lengths of time [54~~3~~]. Due to declining fix rate over time in the WGR herd, we calculated the rolling seven-day average fix rate for each collar and when the average fell below 0.75, we excluded all subsequent days from our analysis.

Fig 3. Seasonal Variation in Herd Structure. Map showing 90% contour from kernel density estimates for elk from the Jackson (JKSN, purple) and West Green River (WGR, blue) herds during low (a,c) and high density (b,d) periods in Wyoming, USA. The location of the National Elk Refuge and Fossil Butte National Monument are indicated by black bordered polygons. The background relief is based on the SRTM 30m digital elevation model provided by NASA JPL [52].

Formatted: Indent: First line: 0"

We calculated pairwise inter-animal distances, daily proximity rates (a measure of the proportion of the day that two animals are within 500 m of each other), and kernel density estimate areas (KDEs) over the full period available for each herd [33]. In previous work [33], we explored other distance thresholds (100 and 250 m) as alternatives to a 500m threshold for daily proximity rates and found comparable relationships across methods. ~~However, a~~At 500 meters, we observed greatest~~r~~ daily fluctuations in proximity rates, which tracked daily changes in abiotic conditions [33]. This indicates that the 500m threshold is more useful for monitoring proximity rates at fine~~r~~ temporal scales within the context of this study system. Evaluating multiple distances ~~is necessary when choosing~~can inform choice of appropriate distance thresholds and may be unique to each study, taxa, spatiotemporal scale, and method or frequency of data collection.

To assess sample size needs, we selected months of lowest and highest densities based on the aggregation metrics. The low- and high-density months included July 2017 (N = 45 elk) and March 2019 (N = 32 elk), respectively for the JKSN herd, and April 2006 (N= 23 elk) and October 2006 (N= 19 elk) for the WGR herd. Because inter-animal distances and proximity rates are daily metrics, we selected the 15th day of each month for our comparison. We summarized pairwise inter-animal distances using the first quartile distance, as this summary is most sensitive to changes in aggregation [43], and we summarized proximity rates using the pairwise mean. We calculated herd-level KDEs and corresponding areas for 50%, 70%, and 90% contours at monthly timescales. We used the default reference bandwidth smoothing parameterization in our KDE estimation, based on the standard deviation of spatial coordinates and sample size [5~~5~~6], to broadly capture the area used by each collared elk population [5~~6~~7]. The smoothing bandwidth can greatly influence how KDEs are drawn, and practitioners should consider their specific

ecological context, project goals, and data structure when calculating KDEs. Our interest centered on the effects of changing sample sizes on metric accuracy rather than fine tuning KDEs for more explicit ecological investigation.

For each herd-month-metric combination, we randomly ~~selected a subset of~~sampled collared elk, incrementing between two and the maximum number of collared elk and resampled 1,000 times with replacement per increment. Assuming the full dataset represented the most accurate value (hereafter baseline value), we assessed variation in aggregation metrics across subsets of collared elk by calculating the percent difference of metrics between the subset and the maximum sample size to determine the accuracy of metrics as sample size increased. For each sample size, we calculated the proportion of subsets within 20% of the baseline value of each aggregation metric. We considered the accuracy threshold to be met when ≥90% of simulations at a given sample size fell within 20% of the baseline value. All aggregation metrics were calculated using the *wildagg* package for program R [44, 5~~7~~8].

Results

The larger JKSN herd typically required larger sample sizes than the smaller WGR herd. Periods when herds were dispersed, with only semi-homogenous and heterogenous mixing (lowest density) required larger minimum sample sizes to achieve accurate measures of all aggregation metrics than periods of homogenous mixing (highest density), regardless of herd identity, (Table 1, Fig 3). During low density months, minimum required sample sizes ranged from 1.9–6.7 times larger than in months of high density for the WGR herd and 1.5–4.2 times larger for the JKSN herd, depending on the aggregation metric (Table 1, Figs 3e-h). To accurately estimate all metrics for the WGR herd, minimum sample sizes of 22 and 16 GPS-collared elk were required under low- and high-density conditions, respectively, which amounts

to 96% and 84% of the maximum available sample size for this herd (Table 1). To accurately estimate all metrics simultaneously for the JKSN herd, sample sizes of 42 and 24 GPS-collared elk were required under low- and high-density conditions, respectively, which reflect 93% and 75% of the maximum available sample size for this herd (Table 1).

Table 1. Minimum sample size required for aggregation metrics

	WGR		JKSN	
Metric	Low Density	High Density	Low Density	High Density
90% KDE Area	13	3	37	11
70% KDE Area	18	3	36	14
50% KDE Area	20	3	36	24
1st Quartile Inter-Animal Distance	21	16	28	14
Proximity rate	21	11	42	10

Minimum sample size required to ensure aggregation metrics are within 20% of the baseline value calculated at maximum available sample size for the Jackson (JKSN) and West Green River (WGR) elk herds in Wyoming, USA during periods of low and high density.

Fig 3. Sample Size Accumulation Curves for Each Aggregation Metric. The proportion of simulations at each sample size that are within 20% of the baseline value calculated at maximum available sample size for elk from the Jackson and West Green River herds in Wyoming, USA. Each aggregation metric is calculated during ~~low~~high- and ~~high~~low-density periods for each herd (a-d, e-h, respectively). Aggregation metrics include the daily 1st quartile inter-elk distance (a,e), mean daily proximity rates (b,f), and the area of 90% (c,g) and 50% contours from kernel density estimates (d,h). Dashed lines indicate when the proportion of simulations within 20% of the baseline value is ≥ 90% (numerical values shown in Table 1). Accumulation curves are fitted using loess smoothing methods for visualization.

Fewer collared elk were needed to produce accurate estimates for KDEs compared to other aggregation metrics for the smaller WGR herd regardless of density conditions (N=3 under high-density and N = 11 under low-density conditions). In contrast, aggregation metrics requiring the most and fewest collared elk to reach the accuracy threshold were more variable for the JKSN herd. Under high density months, 50% KDE area calculations required the most samples and proximity rates required the fewest samples whereas proximity rates and inter-animal distance metrics required the most and fewest samples during periods of low density for the JKSN herd.

As minimum sample size increases under periods of high density for both elk herds, the proportion of simulations within 20% of the baseline value approach 100% well before reaching the maximum available sample size (Figs 3a-d; but see inter-animal distance estimates for the WGR herd in Fig 3a). In contrast, under low density months, the proportion of simulation estimates within 20% of the baseline value of inter-animal distances, proximity rates, and 50% KDE contour area reach 90% within three sample size increments below the maximum available sample size for the WGR herd (Figs 3e-f and 3h). Proximity rate estimates at low density for the JKSN herd was the only metric to not asymptote at higher sample sizes (Fig 3f).

Discussion

Herd size, seasonal density patterns and resulting network complexity modulate the minimum sample size required to accurately estimate aggregation metrics for GPS-collared elk. When elk are widely dispersed during the summer months, the overlap of space use between individuals decreases, requiring a larger sample to capture aggregation patterns for the herd. In contrast, when herds are tightly grouped, as they are on wintering grounds, an individual's space use commonly overlaps with others in the herd. The metrics robust to small sample sizes varied

both within and between herds, indicating that herd context is a crucial consideration when determining the optimum sample size and density metrics for studying aggregation behavior in big game populations.

Our results have considerable implications for how managers can assess disease transmission risk in ungulate herds. Most pressing in western big game populations is the issue of CWD, which has multiple transmission pathways, adding value to consideration of multiple metrics. Chronic wasting disease can be transmitted by direct contact between individuals or indirectly through the contaminated environment (reviewed by [30]). Assessing risk of direct transmission requires approaches like proximity rates (referred to as contact rates in disease ecology), whereas assessing risk of indirect transmission requires estimating overall space use and defining spatial areas of high concentrations which can be achieved through KDE contours. Our analyses indicate proximity rate calculations need more samples due to this metric requiring GPS-collared animals to be within some minimum distance threshold where interactions occur. For demonstration purposes we used a distance threshold of 500m in this analysis, which provided robust estimates for understanding the drivers of elk aggregation in previous work [33], but smaller distance thresholds may be required to capture disease-relevant interactions~~ of individuals likely to transmit disease~~ [5~~8~~9]. As distance thresholds decrease the number of collars in a population will likely need to increase. In contrast, the small sample sizes needed to accurately calculate KDEs likely results from the pooling of location information across individuals to define space use of the herd as a collective.

Transmission of any disease is not uniform across space, time, or from individual to individual [60]. Comparisons such as the one we conducted between herds with different sizes and structure are needed to better understand how transmission risk may differ across unique

populations. Our findings that reasonable accuracy for KDE area, proximity rates and inter-animal distance calculations became obtainable for two distinct herds during two periods of different densities and social structure confirms that GPS collar data can provide accurate aggregation metrics to inform decision-making. Studies predict that Herd density reduction prior to CWD establishment ~~is predicted to~~can result in better long~~-~~-term outcomes (e.g., higher elk abundance) than actions taken after CWD is established in herds~~Recent predictions show that pro-active density management can lead to larger elk numbers than if density is not managed once CWD arrives for a range of elk herd sizes~~ [41-42]. This lends support to multi-agency objectives to use adaptive management approaches targeting ungulate density in various ways to address disease spread [39].

In our study the highest densities and aggregations of elk occur during the winter, when the sexes are together in space and time. In other systems or taxa, the composition of collared individuals (either by sex, age-class, or other characteristics) will need to align with knowledge about transmission dynamics unique to each disease. The mode of transmission (indirect versus direct) will necessarily dictate which aggregations metrics (e.g., KDE for indirect) may be most informative. Researchers should also consider the density characteristics of the population when determining how many collars to deploy and potentially conducting similar resampling analyses as this study presents to fully understand the implications of sampling decisions. This may be especially true for multi-year studies when the opportunity to adjust collar deployments exists. While our results indicate some aggregation metrics can be accurately measured at low sample sizes, ensuring a representative sample of a herd is integral to avoid sampling individuals that differ considerably differ in their spatiotemporal movement patterns thereby inducing biased aggregation estimates. Finally, controlling for collar performance as we have done by censoring

Formatted: Indent: First line: 0.5"

individuals with low fix rates, is integral to avoiding potentially spurious estimates of aggregation.

The aggregation metrics calculated in this study were evaluated within the paradigm of interacting, mobile animals. ~~TAll the metrics evaluated in this study were done under the paradigm of interacting mobile animals.~~ Fixed points on the landscape, for example ~~such as~~ attractants like anthropogenic food sources or mineral licks, can also serve as aggregation hotspots increasing the likelihood of direct contact between individuals and between individuals and a pathogen contaminated environment [30, 61~~59~~]. Using the R package *wildagg* [44], as we did to estimate aggregation metrics, managers could treat ~~also include~~ the locations of attractants as additional 'individuals' to quantify interactions between their wildlife populations and potential point sources of increased pathogen deposition. ~~If~~ When the locations of attractants are unknown, ~~more formal~~ cluster analyses~~, which can be informed by the aggregation metrices explored in this study,~~ may help identify aggregation hotspots [62~~0~~], which can be ~~inform~~described by the aggregation metrics explored in this study ~~[60]~~.

Managers of big game herds rely on aggregation data to make management decisions that can have broad economic and ecological implications such as setting tag quotas, limiting disease transmission risk, or identifying areas with potential for human-wildlife conflict. These results provide researchers and managers with important insight into how many GPS collars should be deployed depending on the size and behavior of their herds. With this knowledge, researchers and managers can direct conservation dollars for greatest return on investment.

Acknowledgements

We thank the Grand Teton Association, U.S. Fish and Wildlife Service, Fossil Butte National Monument, BLM Kemmerer Field Office, Wyoming Game and Fish Department, and

the Bridger-Teton National Forest for supporting GPS collar deployment on the National Elk Refuge and Fossil Butte National Monument. Any use of trade, firm or product names is for descriptive purposes only and does not imply endorsement by the U.S. Government.

Data availability statement

West Green River elk herd GPS data are publicly available and housed on U.S. Geological Survey ScienceBase (https://doi.org/10.5066/F70K27SF) [631]. Jackson elk herd GPS data are available upon request via the National Elk Refuge and Grand Teton National Park (grte_information@nps.gov, attn: Sarah Dewey). Raw aggregation metric data used in this study are available here: https://doi.org/10.5066/P14KSJQM [64].

References

1. Gordon IJ, Hester AJ, Festa-Bianchet MA. The management of wild large herbivores to meet economic, conservation and environmental objectives. Journal of Applied Ecology. 2004 Dec;41(6):1021-31.
2. Robinson KF, Fuller AK, Hurst JE, Swift BL, Kirsch A, Farquhar J, et al. Structured decision making as a framework for large-scale wildlife harvest management decisions. Ecosphere. 2016 Dec;7(12):e01613.
3. U.S. Department of the Interior [U.S. DOI], U.S. Fish and Wildlife Service. National Survey of Fishing, Hunting, and Wildlife-Associated Recreation. 2022 [Cited 2025 Apr 10]. Available from: https://www.fws.gov/media/2022-national-survey-fishing-hunting-and-wildlife-associated-recreation.

4. Jones JD, Proffitt KM, Paterson JT, Almberg ES, Cunningham JA, Loveless KM. Elk responses to management hunting and hazing. The Journal of Wildlife Management. 2021 Nov;85(8):1721-38.
5. Côté SD, Rooney TP, Tremblay JP, Dussault C, Waller DM. Ecological impacts of deer overabundance. Annu. Rev. Ecol. Evol. Syst.. 2004 Dec 15;35(1):113-47.
6. Lambert S, Thébault A, Rossi S, Marchand P, Petit E, Toïgo C, et al. Targeted strategies for the management of wildlife diseases: the case of brucellosis in Alpine ibex. Veterinary Research. 2021 Sep 14;52(1):116.
7. Rabe MJ, Rosenstock SS, deVos Jr JC. Review of big-game survey methods used by wildlife agencies of the western United States. Wildlife Society Bulletin. 2002 Apr 1:46-52.
8. Gaillard JM, Festa-Bianchet M, Yoccoz NG. Population dynamics of large herbivores: variable recruitment with constant adult survival. Trends in ecology & evolution. 1998 Feb 1;13(2):58-63.
9. Storm DJ, Samuel MD, Rolley RE, Shelton P, Keuler NS, Richards BJ, et al. Deer density and disease prevalence influence transmission of chronic wasting disease in white-tailed deer. Ecosphere. 2013 Jan;4(1):1-4.
10. Krausman PR, Bleich VC. Conservation and management of ungulates in North America. International Journal of Environmental Studies. 2013 Jun 1;70(3):372-82.
11. Ferrari MJ, Perkins SE, Pomeroy LW, Bjørnstad ON. Pathogens, social networks, and the paradox of transmission scaling. Interdisciplinary perspectives on infectious diseases. 2011;2011(1):267049.

12. Whitney K. History of wildlife tracking technologies. In Oxford Research Encyclopedia of Environmental Science 2022 Mar 23. https://doi.org/10.1093/acrefore/9780199389414.013.725.

13. Craighead FC, Craighead JJ, Cote CE, Buechner HK. Satellite and ground radiotracking of elk. 1972. Available from: https://ntrs.nasa.gov/citations/19720017418.

14. Hebblewhite M, Haydon DT. Distinguishing technology from biology: a critical review of the use of GPS telemetry data in ecology. Philosophical Transactions of the Royal Society B: Biological Sciences. 2010 Jul 27;365(1550):2303-12.

15. Katzner TE, Arlettaz R. Evaluating contributions of recent tracking-based animal movement ecology to conservation management. Frontiers in Ecology and Evolution. 2020 Jan 24;7:519.

16. Lahoz-Monfort JJ, Magrath MJ. A comprehensive overview of technologies for species and habitat monitoring and conservation. BioScience. 2021 Oct;71(10):1038-62.

17. Tosa MI, Schauber EM, Nielsen CK. Localized removal affects white-tailed deer space use and contacts. The Journal of Wildlife Management. 2017 Jan;81(1):26-37.

18. Cross PC, Creech TG, Ebinger MR, Heisey DM, Irvine KM, Creel S. Wildlife contact analysis: emerging methods, questions, and challenges. Behavioral Ecology and Sociobiology. 2012 Oct;66:1437-47.

19. Cross PC, Lloyd-Smith JO, Bowers JA, Hay CT, Hofmeyr M, Getz WM. Integrating association data and disease dynamics in a social ungulate: bovine tuberculosis in African buffalo in the Kruger National Park. InAnnales Zoologici Fennici 2004 Jan 1 (pp. 879-892). Finnish Zoological and Botanical Publishing Board.

20. Wilber MQ, Yang A, Boughton R, Manlove KR, Miller RS, Pepin KM, et al. A model for leveraging animal movement to understand spatio-temporal disease dynamics. Ecology Letters. 2022 May;25(5):1290-304.

21. Malmberg JL, Alder J, Killion H, Buttke D, Pepin KM, Wittemyer G. Cross–Species Transmission at the Wildlife–Livestock Interface: A case study of epidemiological inference from mule deer GPS collar data. Ecology and Evolution. 2025 Apr;15(4):e71182.

22. Schauber EM, Storm DJ, Nielsen CK. Effects of joint space use and group membership on contact rates among white-tailed deer. The Journal of wildlife management. 2007 Feb;71(1):155-63.

23. Williams DM, Dechen Quinn AC, Porter WF. Informing disease models with temporal and spatial contact structure among GPS-collared individuals in wild populations. PLoS One. 2014 Jan 7;9(1):e84368.

24. Sargeant GA, Wild MA, Schroeder GM, Powers JG, Galloway NL. Spatial network clustering reveals elk population structure and local variation in prevalence of chronic wasting disease. Ecosphere. 2021 Dec;12(12):e03781.

25. Cross PC, Cole EK, Dobson AP, Edwards WH, Hamlin KL, Luikart G, et al. Probable causes of increasing brucellosis in free-ranging elk of the Greater Yellowstone Ecosystem. Ecological Applications. 2010 Jan;20(1):278-88.

26. Proffitt KM, Anderson N, Lukacs P, Riordan MM, Gude JA, Shamhart J. Effects of elk density on elk aggregation patterns and exposure to brucellosis. The Journal of wildlife management. 2015 Apr;79(3):373-83.

27. Wilber MQ, Pepin KM, Campa III H, Hygnstrom SE, Lavelle MJ, Xifara T, et al. Modelling multi-species and multi-mode contact networks: implications for persistence of bovine tuberculosis at the wildlife–livestock interface. Journal of Applied Ecology. 2019 Jun;56(6):1471-81.

28. Richards B. Distribution of chronic wasting disease in North America. United States Geological Survey, National Wildlife Health Center. 2025 [Cited 2025 Mar 4]. Available from: https://www.usgs.gov/media/images/distribution-chronic-wasting-disease-north-america-0.

29. Tranulis MA, Gavier-Widén D, Våge J, Nöremark M, Korpenfelt SL, Hautaniemi M, et al. Chronic wasting disease in Europe: new strains on the horizon. Acta Veterinaria Scandinavica. 2021 Dec;63:1-5.

30. Escobar LE, Pritzkow S, Winter SN, Grear DA, Kirchgessner MS, Dominguez-Villegas E, et al. The ecology of chronic wasting disease in wildlife. Biological Reviews. 2020 Apr;95(2):393-408.

31. Wilson-Henjum G, Ricci L, Stoner DC, Longshore K, Manlove KR. Desert bighorn sheep home range and disease transmission risk responses to temporally dynamic environmental variation. The Journal of Wildlife Management. 2025 Apr;89(3):e22715.

32. Pepin KM, Davis AJ, Beasley J, Boughton R, Campbell T, Cooper SM, et al. Contact heterogeneities in feral swine: implications for disease management and future research. Ecosphere. 2016 Mar;7(3):e01230.

33. Janousek WM, Graves TA, Berman EE, Chong GW, Cole EK, Dewey SR, et al. Human activities and weather drive contact rates of wintering elk. Journal of Applied Ecology. 2021 Mar;58(3):667-76.

34. Jennelle CS, Walter WD, Crawford J, Rosenberry CS, Wallingford BD. Movement of white-tailed deer in contrasting landscapes influences management of chronic wasting disease. The Journal of Wildlife Management. 2022 Nov;86(8):e22306.

35. Janousek WM, Cole EK, Dewey SR, Graves TA. Informing adaptive management to reduce ungulate aggregations: A case study involving winter feeding of elk. Wildlife Society Bulletin. 2025 Mar;49(1):e1574.

36. Sequeira AM, Heupel MR, Lea MA, Eguíluz VM, Duarte CM, Meekan MG, et al. The importance of sample size in marine megafauna tagging studies. Ecological Applications. 2019 Sep;29(6):e01947.

37. Shimada T, Thums M, Hamann M, Limpus CJ, Hays GC, FitzSimmons NN, et al. Optimising sample sizes for animal distribution analysis using tracking data. Methods in Ecology and Evolution. 2021 Feb;12(2):288-97.

38. He P, Klarevas-Irby JA, Papageorgiou D, Christensen C, Strauss ED, Farine DR. A guide to sampling design for GPS-based studies of animal societies. Methods in Ecology and Evolution. 2023 Aug;14(8):1887-905.

39. Western Association of Fish and Wildlife Agencies [WAFWA]. Recommendations for adaptive management of chronic wasting disease in the West. WAFWA Wildlife Health Committee and Mule Deer Working Group. Edmonton, Alberta, Canada and Fort Collins, Colorado, USA. 2017. Available from: https://wafwa.org/wpdm-

package/recommendations-for-adaptive-management-of-chronic-wasting-disease-in-the-west/.

40. Moss WE, Binfet J, Hall LE, Allen SE, Edwards WH, Jennings-Gaines JE, et al. The effectiveness of harvest for limiting wildlife disease: Insights from 20 years of chronic wasting disease in Wyoming. Ecological Applications. 2025 Jan;35(1):e3089.

41. Cook JD, Cross PC. Decision analysis in support of the National Elk Refuge bison and elk management plan: U.S. Geological Survey Scientific Investigations Report 2024–5119; 2025. 5 chap. (A–E), variously paged, https://doi.org/10.3133/sir20245119.

42. Cross PC, Cook JD, Cole, EK. Predictions of elk and chronic wasting disease dynamics at the National Elk Refuge in Jackson, Wyoming, and surrounding areas, chap. B of Cook, JD, and Cross PC, eds., Decision analysis in support of the National Elk Refuge bison and elk management plan: U.S. Geological Survey Scientific Investigations Report 2024–5119; 2025. 22 p., https://doi.org/10.3133/sir20245119B.

43. Graves TA, Yarnall MJ, Johnston AN, Preston TM, Chong GW, Cole EK, et al. Eyes on the herd: Quantifying ungulate density from satellite, unmanned aerial systems, and GPS collar data. Ecological Applications. 2022 Jul;32(5):e2600.

44. Janousek WM, Graves TA. wildagg: Wildlife Aggregation Estimation & Visualization. U.S. Geological Survey software release. Reston, Virginia. Version 0.1.2. 2021. Available from: https://doi.org/10.5066/P9DTQEJS.

45. Wey T, Blumstein DT, Shen W, Jordán F. Social network analysis of animal behaviour: a promising tool for the study of sociality. Animal behaviour. 2008 Feb 1;75(2):333-44.

46. Anderson RM, May RM. Population biology of infectious diseases: Part I. Nature. 1979 Aug 2;280(5721):361-7.

47. Smith MJ, Telfer S, Kallio ER, Burthe S, Cook AR, Lambin X, et al. Host–pathogen time series data in wildlife support a transmission function between density and frequency dependence. Proceedings of the National Academy of Sciences. 2009 May 12;106(19):7905-9.

48. Benson JF, Keiter DA, Mahoney PJ, Allen BL, Allen L, Álvares F, et al. Intrinsic and environmental drivers of pairwise cohesion in wild Canis social groups. Ecology. 2025 Jan;106(1):e4492.

49. Kaur P, Ciuti S, Ossi F, Cagnacci F, Morellet N, Loison A, et al. A protocol for assessing bias and robustness of social network metrics using GPS based radio-telemetry data. Movement Ecology. 2024 Aug 6;12(1):55.

50. Chapman SS, Bryce SA, Omernik JM, Despain DG, ZumBerge J, Conrad M. Ecoregions of Wyoming (color poster with map, descriptive text, summary tables, and photographs): Reston, Virginia, U.S. Geological Survey (map scale 1:1,400,000). 2004. Available from: https://www.epa.gov/eco-research/ecoregion-download-files-state-region-8#pane-48.

~~50.~~51. Buskirk SW, Wild Mammals of Wyoming and Yellowstone National Park. 2016 Univ of California Press.

52. Farr TG, Rosen PA, Caro E, Crippen R, Duren R, Hensley S, et al. The shuttle radar topography mission. Reviews of geophysics. 2007 Jun;45(2).

~~51.~~53. U.S. Census Bureau. Cartographic Boundary File: Current State and Equivalent Entities “cb_2018_us_state_500k”. U.S. Department of Commerce. 2018. https://www.census.gov/geographies/mapping-files/time-series/geo/carto-boundary-file.html.

Formatted: Font: (Default) Times New Roman, 12 pt

~~52.~~54. U.S. Fish and Wildlife Service [USFWS]. Bison and Elk Management Step-down Plan. National Elk Refuge, Grand Teton National Park, Wyoming. Lakewood, CO: U.S. Department of the Interior, U.S. Fish and Wildlife Service. National Park Service. 2019. Available from: https://iris.fws.gov/APPS/ServCat/Reference/Profile/154124.

~~53.~~55. Mikle NL, Graves TA, Olexa EM. To forage or flee: lessons from an elk migration near a protected area. Ecosphere. 2019 Apr;10(4):e02693.

~~54.~~56. Altmann M. Social behavior of elk, *Cervus canadensis nelsoni*, in the Jackson Hole area of Wyoming. Behaviour. 1952 Jan 1:116-43.

~~55.~~57. Worton BJ. Kernel methods for estimating the utilization distribution in home-range studies. Ecology. 1989 Feb;70(1):164-8

~~56.~~58. Gitzen RA, Millspaugh JJ, Kernohan BJ. Bandwidth selection for fixed-kernel analysis of animal utilization distributions. The Journal of Wildlife Management. 2006 Dec;70(5):1334-44.

~~57.~~59. R Core Team. R: A language and environment for statistical computing. R Foundation for Statistical Computing, Vienna, Austria. 2021[cited 2025 Apr 5].

60. Habib TJ, Merrill EH, Pybus MJ, Coltman DW. Modelling landscape effects on density–contact rate relationships of deer in eastern Alberta: implications for chronic wasting disease. Ecological Modelling. 2011 Aug 10;222(15):2722-32.

~~58.~~61. Albery GF, Sweeny AR, Becker DJ, Bansal S. Fine-scale spatial patterns of wildlife disease are common and understudied. Functional Ecology. 2022 Jan;36(1):214-25.

~~59.~~62. Plummer IH, Johnson CJ, Chesney AR, Pedersen JA, Samuel MD. Mineral licks as environmental reservoirs of chronic wasting disease prions. PloS one. 2018 May 2;13(5):e0196745.

~~60.~~63. Cotterill GG, Cross PC, Cole EK, Dewey SR, Wise BL, Graves TA. Ungulate personality and the human shield contribute to long-distance migration loss. BioRxiv. [Preprint]. 2024 [cited 2025 May 15]. Available from: https://doi.org/10.1101/2024.12.10.627781.

64. Graves TA, Mikle NL, Olexa EM. West Green River elk herd locations in southwestern Wyoming, 2005-2010; 2019 [cited 2025 Apr 5]. U.S. Geological Survey data release. Available from: https://doi.org/10.5066/F70K27SF.

~~61.~~65. Janousek WM, Cotterill, GG, Lobo, O, Cole, EK, Dewey, SR, Graves, TA. Estimates of Elk Aggregation Metrics Calculated Across Varying Sample Sizes from Two Herds in Western Wyoming, USA. 2025 [cited 2025 July 23]. US Geological Survey data release https://doi.org/10.5066/P14KSJQM.

Response to Editor

From the second review:

1. **Authorship change –** there was no authorship change during the submission of the manuscript. All authors were appropriately listed on the submitted manuscript document prior to the initial submission. One author was accidentally left out of the authorship tab on the submission platform during the initial submission process and was subsequently added. The original manuscript contained an accurate author contribution statement.

2. **Data availability**
    a. Raw GPS-collar data from elk in the Jackson herd is sensitive due to concerns about potential misuse, particularly related to hunting pressure and poaching. These elk congregate in large numbers within protected landscapes like national parks and wildlife refuges during certain times of the year, making them especially vulnerable if location data is not handled responsibly. Given these factors the collar data is available upon request but continues to be freely shared for research purposes (recent example: Cotterill et al. 2025 *Ecosphere*).
    b. Citations in text and in the data availability statement, including URL's, are provided for the West Green River elk herd GPS data.

3. **Copyright status of map images** – No map products used in this publication are copyrighted. We updated the legend of Figure 2 to identify the sourcing of the inset map (U.S. Census Bureau. Cartographic Boundary File: Current State and Equivalent Entities "cb_2018_us_state_500k". U.S. Department of Commerce. 2018. https://www.census.gov/geographies/mapping-files/time-series/geo/carto-boundary-file.html.)

From the initial review:

General Comments:

While the study addresses an important topic with valuable potential applications, the reviewer has identified critical issues impacting scientific rigor, clarity, generalizability, and practical utility. Addressing these concerns thoroughly—particularly the methodological issues (GPS resolution, sex exclusion, threshold sensitivity), abstract restructuring, seasonal context, disease link clarification, and the addition of a limitations section—is essential for the manuscript to meet publication standards. Major

revision is warranted. Acceptance is contingent on satisfactorily addressing all points raised.

Response:

In our response to reviewer document, we have addressed the line-by-line comments provided by the reviewer covering the topics bulleted here. We argue that many of these points are not limitations of the study but part of the unique considerations that must be made by researchers when conducting similar analyses on animal aggregation with GPS based technologies. To that end we have integrated relevant text throughout the manuscript to reiterate these considerations and have added a paragraph in the discussion to provide readers with guidance on how to relate our findings to their specific research questions.

Specific questions related to Journal Requirements:

1. When submitting your revision, we need you to address these additional requirements.

Please ensure that your manuscript meets PLOS ONE's style requirements, including those for file naming. The PLOS ONE style templates can be found at

https://journals.plos.org/plosone/s/file?id=wjVg/PLOSOne_formatting_sample_main_body.pdf and

https://journals.plos.org/plosone/s/file?id=ba62/PLOSOne_formatting_sample_title_authors_affiliations.pdf

2. Thank you for stating the following financial disclosure:

Funding was provided by the U.S. Geological Survey Biothreats Program.

Please state what role the funders took in the study. If the funders had no role, please state: "The funders had no role in study design, data collection and analysis, decision to publish, or preparation of the manuscript."

If this statement is not correct you must amend it as needed.

Please include this amended Role of Funder statement in your cover letter; we will change the online submission form on your behalf.

We have responded to this question in the cover letter as requested. The funders had no role in the study.

3. We noted in your submission details that a portion of your manuscript may have been presented or published elsewhere. “Some of the raw data used in this analysis has been previously published in a data repository and is cited in text”. Please clarify whether this [conference proceeding or publication] was peer-reviewed and formally published. If this work was previously peer-reviewed and published, in the cover letter please provide the reason that this work does not constitute dual publication and should be included in the current manuscript.

We agree this statement is confusing. Our intention was to acknowledge the source of the West Green River elk herd GPS data, which was published prior to this manuscript on a USGS’s public data repository, ScienceBase. All work in this manuscript is original although some of the GPS data has been used in other studies. One study examined the biological drivers of aggregation in the Jackson elk herd (Janousek et al. 2021). A second study examined the differences in density estimates across GPS, satellite imagery, and drone counting methods for the Jackson elk herd (Graves et al. 2022). This 2025 study explores the accuracy of aggregation metrics derived from varying GPS collar sample sizes, focuses on multiple and different time periods than the other work, and is a novel addition/extension of the previous works.

4. In the online submission form, you indicated that your data is available only on request from a third party. Please note that your Data Availability Statement is currently missing the contact details for the third party, such as an email address or a link to where data requests can be made. Please update your statement with the missing information.

We added an email contact for readers regarding the requestable data (grte_information@nps.gov, attn: Sarah Dewey)

5. Thank you for uploading your study's underlying data set. Unfortunately, the repository you have noted in your Data Availability statement does not qualify as an acceptable data repository according to PLOS's standards.

At this time, please upload the minimal data set necessary to replicate your study's findings to a stable, public repository (such as figshare or Dryad) and provide us with the relevant URLs, DOIs, or accession numbers that may be used to access these data. For a list of recommended repositories and additional information on PLOS standards for data deposition, please see https://journals.plos.org/plosone/s/recommended-repositories.

The U.S. Geological Service’s online data repository, ScienceBase, is commonly used and follows best practices for data storage and availability for USGS-related

projects. For example, Sanosm et al. 2024, Baldwin et al. 2020, Orzechowski et al. 2019 ; recent PLOS One published studies.

We have provided accessibility options for the raw data used in this study and we have also now published the aggregation metrics dataset from which we made our assessments. This new dataset is available on USGS ScienceBase. All USGS ScienceBase products have a stable URL, DOI, and undergo extensive quality review. We've updated the 'Data Availability' statement with this new information and all data citations in References include DOIs.

6. Please amend the manuscript submission data (via Edit Submission) to include author Dr. Sarah R. Dewey.

Done

7. We note that Figure 2 in your submission contain map images which may be copyrighted. All PLOS content is published under the Creative Commons Attribution License (CC BY 4.0), which means that the manuscript, images, and Supporting Information files will be freely available online, and any third party is permitted to access, download, copy, distribute, and use these materials in any way, even commercially, with proper attribution. For these reasons, we cannot publish previously copyrighted maps or satellite images created using proprietary data, such as Google software (Google Maps, Street View, and Earth). For more information, see our copyright guidelines: http://journals.plos.org/plosone/s/licenses-and-copyright.

The background imagery used in Figures 2 and 3 is an elevation layer publicly available and the source is cited in the figure caption. We added additional language for increased clarity: "*The background relief is based on the SRTM 30m digital elevation model provided by NASA JPL (Farr et al. 2007).*" No copyrighted material is contained in any figure. We use the elevation data source's recommended citation for this data: Farr TG, Rosen PA, Caro E, Crippen R, Duren R, Hensley S, et al. The shuttle radar topography mission. Reviews of geophysics. 2007 Jun;45(2).

8. If the reviewer comments include a recommendation to cite specific previously published works, please review and evaluate these publications to determine whether they are relevant and should be cited. There is no requirement to cite these works unless the editor has indicated otherwise.

Done

Response to Reviewer

Reviewer #1: Line 20 – 24: The general background written doesn't have continuity with the research statement given. It would be better if the authors start the abstract with a general background on "social aggregation in ungulates, its basic importance (e.g., it helps in shaping behavioral dynamics, and resource use), and how it can lead to infectious disease transmission. This should be followed by introducing the "Ungulate herd density metrics" and why it is important in understanding social aggregation in terms of disease risk and transmission, like chronic wasting disease (CWD).

We've revised the abstract following the reviewer's suggestions.

Lines 26-28: This identifies the research gaps, which should be followed by the above statement when revised.

Done.

Line 30-32: How many years of data were collected? The study area should be mentioned in the abstract

Done.

Line 41-43: In terms of management goals, the author should be specific about what goals we are talking about. Whether it is related to only active management of herds, or any other measures that reduce the disease transmission among populations.

We argue that using the general term 'management goals' is sufficient for the abstract considering word limit requirements and given that we elaborate in more detail on various management objectives and activities elsewhere in the manuscript.

Line 63-64: "The proliferation of tracking……." can be revised to "The proliferation of tracking technology has emerged as a valuable tool to meet this need by enabling detailed monitoring of animal movements."

We incorporated your suggestion, thanks.

Line 65: Delete "purpose"

Done

Line 67 – 69: Is the reference used here specific to elk or in general?? I suggest adding more recent references specific to elk that justify your statement.

Two references are cited in this sentence one supporting the understanding that elk were the first species tracked using satellite technology (Craighead et al.

1972) and the second reference is a well-cited review of the broad adoption GPS telemetry in ecological studies (Hebblewhite and Haydon 2010).

Line 70 – 71: Elaborate on the costly operations that can cause stress or risk to the animal studied.

We added more context to this statement: While methods for capturing and handling ungulates have improved, these are still *financially* costly *operations (e.g., helicopter flights, weeks of staff time)* that can induce stress or risk *injuring* animal subjects [15-16].

Lines 72 – 75: For example, proximity sensors are placed on the GPS/GSM/VHF collars that detect proximity between animals or between animals and other species, providing data on interaction rates and contact zones.

We have taken your suggestions and edited this statement: More recently, *the addition of* proximity sensors to collars *has emerged as a promising tool* capable of recording pairwise *interaction rates among* individuals, but these too, require direct capture and handling for deployment [17].

Line 85: Explain to the readers how location data can be used to predict disease transmission, and about the tools.

We edited this statement clarifying our intent – that GPS location data " …permit the analysis of movement paths, conspecific contact rates, and spatiotemporal density patterns *informing* disease transmission risk…"

Lines 102-103: The manuscript focuses on the social aggregation of herds and suggests measures to reduce the cost associated with understanding aggregation metrics. Lines 102-103 talk about the cost reduction and personal hours. I suggest writing more information to give a complete picture to the readers, from ecological to economic benefits.

There's a limit to what we can say with respect to any one particular management agency's resources allocated for research or collaring studies. We leave this to managers to evaluate based on their own costs and resource constraints. The ecological benefit of deploying collars is derived from the information they provide – here we largely focus on the utility of collar data to specifically inform the ecology of disease. To that end we seek to develop better methods for analyzing this data which can inform ecological research. We have edited Lines 76-91 to more clearly speak these costs/benefits.

Line 117: The reference cited to support the statement should be across different taxa. Include more references that have studied social behavior among ungulates or other taxa.

We edited this statement for clarity and added additional citations.

Line 118: The comments for Lines 70 – 71 should support this opening statement; that's how this study is important in terms of ecological and economic values.

See previous responses where this section is addressed specifically.

Line 145 and 146: What are the values inside the parentheses, (NER 43.4805, -110.7428) and (FOBU, 41.8558, -110.7615)?

The values represent latitude and longitude in decimal degrees. We've added 'N' and 'W' to indicate this more clearly. E.g., NER 43.4805 N, -110.7428 W.

Line 160: The figure itself is not sufficient to explain the study area. Firstly, I suggest adding the state/country subset map highlighting the study area location. Second, for both the locations, i.e, JKSN and WGR, figure 2 (a) and (c) show the summer conditions while (b) and (d) show winter aggregations. This should appear in the map as summer and winter range, and be included in the figure title as well.

We have taken this advice and created a separate figure for the general study area showing the state of Wyoming, where it falls within the United States, and the specific locations of the two study areas (now Figure 2). We also added the seasonal periods (winter vs summer) to the titles of the subplots in Figure 3 as requested (originally Figure 2).

Line 177 - 203:
- There appears to be a potential difference in GPS collar resolution across the two study herds (JKSN and WGR). How do the authors account for fix frequency when comparing daily aggregation metrics like inter-animal distance and proximity rates? Could coarser data bias metric estimation?

Coarser resolution data could bias metric estimates when it comes to rate of successful fixes which we account for by censoring individuals with lower fix rates (see Lines 191-197). This is also now reiterated in the discussion (Lines 339-341).

-Given that proximity rates and first quartile distances are highly sensitive to fix rate and temporal precision, how accurate are these metrics in periods or individuals with coarser-scale GPS data? Have the authors conducted any sensitivity analysis to evaluate metric performance under lower-resolution scenarios?

See previous response as well. Daily metrics estimated with coarser resolution data should decrease the precision of the estimate but not necessarily the accuracy. We indicate in the materials and methods how we strive to control for

factors that could influence the sensitivity of the metrics we estimated (Lines 191-197).

-Why were only female elk included in the analyses? Were there male individuals collared during the study period, and if so, what was the rationale for excluding them? The sex-specific social behavior in ungulates could influence aggregation metrics.

Males were not excluded per se, rather only females are typically collared at large numbers in these populations. This is less of an issue in this study when the period of highest elk density involves high overlap in space use across the sexes. However, in other disease contexts or taxa sex-specific differences could be important to consider. See additional response below on this topic and new text added to discussion (Lines 327-341).

-The authors chose a 500 m threshold for defining proximity events and the first quartile of pairwise distances to summarize aggregation. Can the authors justify these thresholds ecologically? Was there any sensitivity analysis performed to explore how changing these values affects aggregation estimates?

We have updated the Methods section to include more detail regarding the use of 500m distance thresholds for proximity rates. " ***In previous work [33], we explored other distance thresholds (100 and 250 m) as alternatives to a 500m threshold for daily proximity rates and found comparable relationships across methods. However, at 500 meters, we observed greater daily fluctuations in proximity rates, which tracked daily changes in abiotic conditions [33]. This indicates that the 500m threshold is more useful for monitoring proximity rates at fine temporal scales within the context of this study system. Evaluating multiple distances is necessary when choosing appropriate distance thresholds and may be unique to each study, taxa, spatiotemporal scale, and method or frequency of data collection."***

-The KDEs were generated using default reference bandwidths without fine-tuning. Could this choice, particularly under varying sample sizes and spatial dispersions, influence the comparability or accuracy of density estimates? This could either over/underestimate the home ranges. If the individuals follow range residency, was any analysis carried out to see their semivariograms? For example, the estimates would vary for home ranges when autocorrelation is accounted for while estimating home ranges.

We point out on Lines 224-230 that selecting, or fine-tuning, the bandwidths informing KDEs can influence their areas. Importantly, we use the same settings across herds to evaluate differences between herds and seasons. Like our other metrics, consistency is critical to make meaningful comparisons. Our emphasis is not on the 'correct' assessment of individual home ranges but rather space use of the herd as a collective (reiterated on Line 223). While individual home ranges are important in other contexts, for instance in resource selection studies, in this study it is the relative, not absolute, size of the estimator that matters. Given that

framing, semivariograms make less sense in the herd context as these are typically calculated for individual movement paths to assess whether animals are settled on a home range or otherwise assess spatial autocorrelation.

Line 209-210: The authors report a wide range in minimum required sample sizes (1.9 – 6.7) for WGR and (1.5 – 4.2) JKSN, between low and high density months. Could this variability be an artifact of differences in resolution of GPS fixes between herds or across time? Were collars across sites and years standardized in fix interval, and if not, how might temporal irregularity influence these estimations?

We appreciate the reviewer's attention to detail and assumptions. Resolution of GPS fixes were stable across time (years or seasons within years) so the within herd variation is not due to fix rate changing over time. We also exclude individuals whose collars exhibited high rates of missed fixes from the analysis (Lines 191-197). Our results suggest the variation in minimum sample sizes is driven by the different densities of the herds, their grouping structures (homogenous to heterogenous mixing) across seasons, and the ability of the metrics to capture aggregation across these contexts (Lines 288-296).

Line 219: The metrics given in the table were tested for robustness under non-random sampling conditions, such as when subsamples included or excluded spatially central vs. peripheral individuals? How might the spatial location of individuals within a herd influence metric estimation bias under small sample sizes?

Sampling was random in our analysis. Lines 231-240: *"For each herd-month-metric combination, we randomly selected a subset of collared elk, incrementing between two and the maximum number of collared elk and resampled 1,000 times with replacement per increment."* The variation of space use by individuals is what drives the variation KDE estimates as sample size changes. The reason for the 1,000 resamples taken at each sample size increment to capture that variation. However, at low sample sizes, the chance sampling of individuals that differ considerably in patterns of space use could induce bias in aggregation metrics – or make them challenging to estimates – and as the reviewer points out here this is worth noting in the implications section of the discussion – Lines 336-339 now includes: *"While our results indicate some aggregation metrics can be accurately measured at low sample sizes, ensuring a representative sample of a herd is integral to avoid sampling individuals that differ considerably differ in their spatiotemporal movement patterns thereby inducing biased aggregation estimates."*

Line 224: In Figure 3, the manuscript text refers to panels a–h; however, these panel labels are not present in the figure itself. Please revise the figure to include clear and consistent panel labels (e.g., a–h) to match the references in the main text. This will improve clarity and ensure accurate interpretation of the results

Thanks for catching this. Done. This figure is now Figure 4.

Line 242: The proximity rate metric for the JKSN and WGR, herd under low-density conditions, did not asymptote with increasing sample size. Could the authors clarify whether this is due to behavioral heterogeneity (e.g., subgrouping or fission–fusion dynamics) or again a function of resolution or inconsistent temporal overlap among individuals?

Metrics failing to asymptote with increasing sample size under certain conditions may indicate that the number of collars deployed in a particular herd and time was insufficient to capture the true aggregation. This is less of an issue for our study where our main interest is how similar the aggregation metrics estimated from subsamples of collars are to the estimates from the full sample size. However, it does highlight the need for managers or researchers to critically consider the density characteristics of the herd when determining how many collars to deploy on a herd and potentially conducting similar resampling analyses as this study presents to fully understand. We reiterate this on Lines 332-335.

Line 251-259: The discussion does not acknowledge the potential effect of irregular GPS collar fix rates or temporal resolutions across sites. Could differences in data resolution, not just herd size or seasonal density, have contributed to variation in required sample sizes? Please clarify if collar performance was controlled for in this interpretation.

Collar performance was controlled for in this study. See previous comment above on the irregular GPS collar fix rates. Resolution of GPS fixes were stable across time (years or seasons within years) so the within herd variation is not due to fix rate changing over time. We also excluded individuals whose collars exhibited high rates of missed fixes from the analysis (Lines 191-197).

Lines 260–273: The discussion draws broad conclusions about disease transmission risk and aggregation behavior without acknowledging the study's restriction to only female elk. Given that males may exhibit different space use and social structures, how might these sex-based exclusions limit the generalizability of the findings to population-wide disease management?

We disagree that we draw broad conclusions about disease transmission risk, rather we cite other studies that have shown how host density and aggregations patterns influence pathogen transmission. In general, elk are highly social, and although they can exhibit sexual segregation (particularly during summer), their overall space-use patterns are comparable. Though we would like to have been able to include male data, it is common in elk studies that few, or no, male elk are included based on the safety risks associated with capturing antlered animals. The most salient point here is that the highest densities and aggregations of elk occur during the winter, when the sexes are together in space and time. This is

also the period where transmission risk is highest for many pathogens affecting elk. In other systems or taxa, the composition of collared individuals will need to be adjusted to align with knowledge about transmission specific to other diseases. We have added text to outline the implications identified here (Lines 327-341).

Lines 270–273: The authors used a 500 m threshold for proximity rates, but note that smaller thresholds may be more relevant for disease transmission. Was any sensitivity analysis performed to evaluate how different thresholds affect sample size needs or proximity estimates? Clarifying this would improve the utility of your findings for applied disease ecology.

We updated the Methods section to provide more clarity on this topic (Lines 207-215). Please see refer to more detailed response above.

Lines 274–276: While KDEs appear robust at low sample sizes, this may be due to the spatial pooling of data. How do the authors reconcile this with the need to capture fine-scale movement or individual-level variation in contact risk?

This point speaks to the nuance of disease transmission. Diseases can differ in transmission mode, direct (animal to animal via contact) or indirect (animal to contaminated environment), and the metrics best suited to answer these questions are also different. KDE's do well in describing the space use of the herd which can highlight areas of increased environmental contamination while proximity rates more directly measure contact between individuals. These points are reiterated in the discussion on Lines 297-313. The metric chosen by a manager to understand aggregation is necessarily tied to the transmission dynamics of the disease in question. We have added text to outline the implications identified here (Lines 330-332).

Lines 288–296: The authors suggest incorporating landscape features like mineral licks as pseudo-individuals for aggregation analysis. How feasible is this with current data, especially if collar resolution or location accuracy is coarse? Can the authors elaborate on how resolution affects the ability to detect point-based aggregation patterns? This should be included in the limitations.

We are not proposing to do this with the current dataset, per se, and collar resolution does play an important role as you point out. Line 342 has been modified to read: "Fixed points on the landscape, for example attractants like anthropogenic food sources or mineral licks, can…" In another recent manuscript, which we cite, these focal points are detectable using GPS collar data.

Other comments:
-Seasonal variation in herd dynamics could be introduced in the introduction of the manuscript. Given that herd structure and aggregation patterns vary substantially

between summer and winter, and this variation underpins key differences in sample size requirements and aggregation metrics, seasonal dynamics should be introduced in the Introduction. Presenting this context early would help readers better understand the ecological drivers of the study and set the stage for subsequent analyses.

We agree that this is an important topic. We introduce seasonal variation in the introduction beginning Line 94 and more extensible Lines 132-148, and even highlight the conceptual significance of these patterns using Figure 1 (also in the introduction): "*Relative ungulate herd densities and contact probabilities fluctuate seasonally (e.g., summer versus winter) with changing biotic and abiotic conditions [33]. During winter, elk herds concentrate on relatively small winter ranges where individuals interact with more conspecifics (homogenous mixing; Fig 1a). During summer, elk disperse across the landscape which reduces relative densities, and results in fewer conspecific contacts (heterogeneous mixing; Figs 1b-c).*" We also further elaborate on the seasonal contexts related to this specific study in the Materials and Methods section Lines 185-190.

- the seasons and differences in vegetation and landscape structure should be clearly written in the study area section in methods.
We provide a comprehensive overview on of the biophysical contexts each herd experiences including topics such as vegetation composition, topographic landscape, elevation, and predator communities. See Lines 158-175: *"The JKSN herd inhabit the Middle Rockies ecoregion defined by steep mountains dominated by coniferous forest and shrub- and grass-covered foothills (1,500–3,000m elevation [50]). During this study, elk from the JKSN herd were observed within an 1,822 km2 area encompassing their wintering grounds on NER and summer range in the Teton and Gros Ventre Ranges, including portions of Grand Teton and Yellowstone National Parks to the west and north and Bridger Teton National Forest to the east (Fig 2). WGR elk inhabit the foothill shrublands and low conifer-dotted mountains of the Wyoming Basin ecoregion (1,500–2,200m elevation [50]). Elk from the WGR herd were observed within a 730 km2 area, including their winter range on Fossil Butte National Monument and summer range in the surrounding Tunp Range which mainly consists of Bureau of Land Management lands (Fig 2). While both herds experience predation risk from mountain lions (Puma concolor), the JKSN herd also live within the range of grizzly bears (Ursus arctos) and gray wolves (Canis lupus)."*

-Given the known differences in elk behavior and group structure between summer and winter, more explicit discussion is needed on how seasonal context influences metric sensitivity, contact rates, and implications for disease management.
We discuss the connections highlighted by the reviewer throughout the discussion:
Lines 290-296: *"When elk are widely dispersed during the summer months, the overlap of space use between individuals decreases, requiring a larger sample to capture aggregation patterns for the herd. In contrast, when herds are tightly grouped, as they are on wintering grounds, an individual's space use commonly*

overlaps with others in the herd. The metrics robust to small sample sizes varied both within and between herds, indicating that herd context is a crucial consideration when determining the optimum sample size and density metrics for studying aggregation behavior in big game populations."

-Please include a brief discussion of the study's limitations. Specifically, address potential effects of varying GPS collar resolution, the exclusive use of female elk, the fixed 500 m proximity threshold, and the absence of sensitivity analyses for sampling design. Acknowledging these will provide important context for interpreting and applying the results

We have added text to expand on the points the reviewer has raised here. Refer to new paragraph in discussion beginning on Line 327 and other changes throughout the manuscript. We respond to each to these points specifically where they appear in the review comments above.
