# Supplementary material for: Estimating GPS-based social aggregation metrics using collar data
Source: PLoS One. 2026 Apr 9;21(4):e0345546. doi: 10.1371/journal.pone.0345546 (PMC13065019; doi:10.1371/journal.pone.0345546)
Supplement: Attachment — Submitted filename: Response to Reviewer_PONE-D-25-26403.docx [file pone.0345546.s001.docx]

**Response to Reviewer**

**Reviewer #1:** Line 20 – 24: The general background written doesn’t have continuity with the research statement given. It would be better if the authors start the abstract with a general background on “social aggregation in ungulates, its basic importance (e.g., it helps in shaping behavioral dynamics, and resource use), and how it can lead to infectious disease transmission. This should be followed by introducing the “Ungulate herd density metrics” and why it is important in understanding social aggregation in terms of disease risk and transmission, like chronic wasting disease (CWD).

**We’ve revised the abstract following the reviewer’s suggestions.**

Lines 26-28: This identifies the research gaps, which should be followed by the above statement when revised.

**Done.**

Line 30-32: How many years of data were collected? The study area should be mentioned in the abstract

**Done.**

Line 41-43: In terms of management goals, the author should be specific about what goals we are talking about. Whether it is related to only active management of herds, or any other measures that reduce the disease transmission among populations.

**We argue that using the general term ‘management goals’ is sufficient for the abstract considering word limit requirements and given that we elaborate in more detail on various management objectives and activities elsewhere in the manuscript.**

Line 63-64: “The proliferation of tracking…….” can be revised to “The proliferation of tracking technology has emerged as a valuable tool to meet this need by enabling detailed monitoring of animal movements.”

**We incorporated your suggestion, thanks.**

Line 65: Delete “purpose”

**Done**

Line 67 – 69: Is the reference used here specific to elk or in general?? I suggest adding more recent references specific to elk that justify your statement.

**Two references are cited in this sentence one supporting the understanding that elk were the first species tracked using satellite technology (Craighead et al. 1972) and the second reference is a well-cited review of the broad adoption GPS telemetry in ecological studies (Hebblewhite and Haydon 2010).**

Line 70 – 71: Elaborate on the costly operations that can cause stress or risk to the animal studied.

**We added more context to this statement: While methods for capturing and handling ungulates have improved, these are still *financially* costly *operations (e.g., helicopter flights, weeks of staff time)* that can induce stress or risk *injuring* animal subjects [15-16].**

Lines 72 – 75: For example, proximity sensors are placed on the GPS/GSM/VHF collars that detect proximity between animals or between animals and other species, providing data on interaction rates and contact zones.

**We have taken your suggestions and edited this statement: More recently, *the addition of* proximity sensors to collars *has emerged as a promising tool* capable of recording pairwise *interaction rates* *among* individuals, but these too, require direct capture and handling for deployment [17].**

Line 85: Explain to the readers how location data can be used to predict disease transmission, and about the tools.

**We edited this statement clarifying our intent – that GPS location data “ …permit the analysis of movement paths, conspecific contact rates, and spatiotemporal density patterns *informing* disease transmission risk…”**

Lines 102-103: The manuscript focuses on the social aggregation of herds and suggests measures to reduce the cost associated with understanding aggregation metrics. Lines 102-103 talk about the cost reduction and personal hours. I suggest writing more information to give a complete picture to the readers, from ecological to economic benefits.

**There’s a limit to what we can say with respect to any one particular management agency’s resources allocated for research or collaring studies. We leave this to managers to evaluate based on their own costs and resource constraints. The ecological benefit of deploying collars is derived from the information they provide – here we largely focus on the utility of collar data to specifically inform the ecology of disease. To that end we seek to develop better methods for analyzing this data which can inform ecological research. We have edited Lines 76-91 to more clearly speak these costs/benefits.**

Line 117: The reference cited to support the statement should be across different taxa. Include more references that have studied social behavior among ungulates or other taxa.

**We edited this statement for clarity and added additional citations.**

Line 118: The comments for Lines 70 – 71 should support this opening statement; that's how this study is important in terms of ecological and economic values.

**See previous responses where this section is addressed specifically.**

Line 145 and 146: What are the values inside the parentheses, (NER 43.4805, -110.7428) and (FOBU, 41.8558, -110.7615)?

**The values represent latitude and longitude in decimal degrees. We’ve added ‘N’ and ‘W’ to indicate this more clearly. E.g., NER 43.4805 N, -110.7428 W.**

Line 160: The figure itself is not sufficient to explain the study area. Firstly, I suggest adding the state/country subset map highlighting the study area location. Second, for both the locations, i.e, JKSN and WGR, figure 2 (a) and (c) show the summer conditions while (b) and (d) show winter aggregations. This should appear in the map as summer and winter range, and be included in the figure title as well.

**We have taken this advice and created a separate figure for the general study area showing the state of Wyoming, where it falls within the United States, and the specific locations of the two study areas (now Figure 2). We also added the seasonal periods (winter vs summer) to the titles of the subplots in Figure 3 as requested (originally Figure 2).**

Line 177 - 203:
- There appears to be a potential difference in GPS collar resolution across the two study herds (JKSN and WGR). How do the authors account for fix frequency when comparing daily aggregation metrics like inter-animal distance and proximity rates? Could coarser data bias metric estimation?

**Coarser resolution data could bias metric estimates when it comes to rate of successful fixes which we account for by censoring individuals with lower fix rates (see Lines 191-197).** **This is also now reiterated in the discussion (Lines 339-341).**

-Given that proximity rates and first quartile distances are highly sensitive to fix rate and temporal precision, how accurate are these metrics in periods or individuals with coarser-scale GPS data? Have the authors conducted any sensitivity analysis to evaluate metric performance under lower-resolution scenarios?

**See previous response as well. Daily metrics estimated with coarser resolution data should decrease the precision of the estimate but not necessarily the accuracy. We indicate in the materials and methods how we strive to control for factors that could influence the sensitivity of the metrics we estimated (Lines 191-197).**

-Why were only female elk included in the analyses? Were there male individuals collared during the study period, and if so, what was the rationale for excluding them? The sex-specific social behavior in ungulates could influence aggregation metrics.

**Males were not excluded per se, rather only females are typically collared at large numbers in these populations. This is less of an issue in this study when the period of highest elk density involves high overlap in space use across the sexes. However, in other disease contexts or taxa sex-specific differences could be important to consider. See additional response below on this topic and new text added to discussion (Lines 327-341).**
-The authors chose a 500 m threshold for defining proximity events and the first quartile of pairwise distances to summarize aggregation. Can the authors justify these thresholds ecologically? Was there any sensitivity analysis performed to explore how changing these values affects aggregation estimates?

**We have updated the Methods section to include more detail regarding the use of 500m distance thresholds for proximity rates. “ *In previous work [33], we explored other distance thresholds (100 and 250 m) as alternatives to a 500m threshold for daily proximity rates and found comparable relationships across methods. However, at 500 meters, we observed greater daily fluctuations in proximity rates, which tracked daily changes in abiotic conditions [33]. This indicates that the 500m threshold is more useful for monitoring proximity rates at fine temporal scales within the context of this study system. Evaluating multiple distances is necessary when choosing appropriate distance thresholds and may be unique to each study, taxa, spatiotemporal scale, and method or frequency of data collection.”***

-The KDEs were generated using default reference bandwidths without fine-tuning. Could this choice, particularly under varying sample sizes and spatial dispersions, influence the comparability or accuracy of density estimates? This could either over/underestimate the home ranges. If the individuals follow range residency, was any analysis carried out to see their semivariograms? For example, the estimates would vary for home ranges when autocorrelation is accounted for while estimating home ranges.

**We point out on Lines 224-230 that selecting, or fine-tuning, the bandwidths informing KDEs can influence their areas. Importantly, we use the same settings across herds to evaluate differences between herds and seasons. Like our other metrics, consistency is critical to make meaningful comparisons. Our emphasis is not on the ‘correct’ assessment of individual home ranges but rather space use of the herd as a collective (reiterated on Line 223). While individual home ranges are important in other contexts, for instance in resource selection studies, in this study it is the relative, not absolute, size of the estimator that matters. Given that framing, semivariograms make less sense in the herd context as these are typically calculated for individual movement paths to assess whether animals are settled on a home range or otherwise assess spatial autocorrelation.**

Line 209-210: The authors report a wide range in minimum required sample sizes (1.9 – 6.7) for WGR and (1.5 – 4.2) JKSN, between low and high density months. Could this variability be an artifact of differences in resolution of GPS fixes between herds or across time? Were collars across sites and years standardized in fix interval, and if not, how might temporal irregularity influence these estimations?

**We appreciate the reviewer’s attention to detail and assumptions. Resolution of GPS fixes were stable across time (years or seasons within years) so the within herd variation is not due to fix rate changing over time. We also exclude individuals whose collars exhibited high rates of missed fixes from the analysis (Lines 191-197). Our results suggest the variation in minimum sample sizes is driven by the different densities of the herds, their grouping structures (homogenous to heterogenous mixing) across seasons, and the ability of the metrics to capture aggregation across these contexts (Lines 288-296).**

Line 219: The metrics given in the table were tested for robustness under non-random sampling conditions, such as when subsamples included or excluded spatially central vs. peripheral individuals? How might the spatial location of individuals within a herd influence metric estimation bias under small sample sizes?

**Sampling was random in our analysis. Lines 231-240: *“For each herd-month-metric combination, we randomly selected a subset of collared elk, incrementing between two and the maximum number of collared elk and resampled 1,000 times with replacement per increment.”*  The variation of space use by individuals is what drives the variation KDE estimates as sample size changes. The reason for the 1,000 resamples taken at each sample size increment to capture that variation. However, at low sample sizes, the chance sampling of individuals that differ considerably in patterns of space use could induce bias in aggregation metrics – or make them challenging to estimates – and as the reviewer points out here this is worth noting in the implications section of the discussion – Lines 336-339 now includes: *“While our results indicate some aggregation metrics can be accurately measured at low sample sizes, ensuring a representative sample of a herd is integral to avoid sampling individuals that differ considerably differ in their spatiotemporal movement patterns thereby inducing biased aggregation estimates.”***

Line 224: In Figure 3, the manuscript text refers to panels a–h; however, these panel labels are not present in the figure itself. Please revise the figure to include clear and consistent panel labels (e.g., a–h) to match the references in the main text. This will improve clarity and ensure accurate interpretation of the results

**Thanks for catching this.** **Done.** **This figure is now Figure 4.**

Line 242: The proximity rate metric for the JKSN and WGR, herd under low-density conditions, did not asymptote with increasing sample size. Could the authors clarify whether this is due to behavioral heterogeneity (e.g., subgrouping or fission–fusion dynamics) or again a function of resolution or inconsistent temporal overlap among individuals?

**Metrics failing to asymptote with increasing sample size under certain conditions may indicate that the number of collars deployed in a particular herd and time was insufficient to capture the true aggregation. This is less of an issue for our study where our main interest is how similar the aggregation metrics estimated from subsamples of collars are to the estimates from the full sample size. However, it does highlight the need for managers or researchers to critically consider the density characteristics of the herd when determining how many collars to deploy on a herd and potentially conducting similar resampling analyses as this study presents to fully understand. We reiterate this on Lines 332-335.**

Line 251-259: The discussion does not acknowledge the potential effect of irregular GPS collar fix rates or temporal resolutions across sites. Could differences in data resolution, not just herd size or seasonal density, have contributed to variation in required sample sizes? Please clarify if collar performance was controlled for in this interpretation.

**Collar performance was controlled for in this study. See previous comment above on the irregular GPS collar fix rates. Resolution of GPS fixes were stable across time (years or seasons within years) so the within herd variation is not due to fix rate changing over time. We also excluded individuals whose collars exhibited high rates of missed fixes from the analysis (Lines 191-197).**

Lines 260–273: The discussion draws broad conclusions about disease transmission risk and aggregation behavior without acknowledging the study’s restriction to only female elk. Given that males may exhibit different space use and social structures, how might these sex-based exclusions limit the generalizability of the findings to population-wide disease management?

**We disagree that we draw broad conclusions about disease transmission risk, rather we cite other studies that have shown how host density and aggregations patterns influence pathogen transmission. In general, elk are highly social, and although they can exhibit sexual segregation (particularly during summer), their overall space-use patterns are comparable. Though we would like to have been able to include male data, it is common in elk studies that few, or no, male elk are included based on the safety risks associated with capturing antlered animals. The most salient point here is that the highest densities and aggregations of elk occur during the winter, when the sexes are together in space and time. This is also the period where transmission risk is highest for many pathogens affecting elk. In other systems or taxa, the composition of collared individuals will need to be adjusted to align with knowledge about transmission specific to other diseases. We have added text to outline the implications identified here (Lines 327-341).**

Lines 270–273: The authors used a 500 m threshold for proximity rates, but note that smaller thresholds may be more relevant for disease transmission. Was any sensitivity analysis performed to evaluate how different thresholds affect sample size needs or proximity estimates? Clarifying this would improve the utility of your findings for applied disease ecology.

**We updated the Methods section to provide more clarity on this topic (Lines 207-215). Please see refer to more detailed response above.**

Lines 274–276: While KDEs appear robust at low sample sizes, this may be due to the spatial pooling of data. How do the authors reconcile this with the need to capture fine-scale movement or individual-level variation in contact risk?

**This point speaks to the nuance of disease transmission. Diseases can differ in transmission mode, direct (animal to animal via contact) or indirect (animal to contaminated environment), and the metrics best suited to answer these questions are also different. KDE’s do well in describing the space use of the herd which can highlight areas of increased environmental contamination while proximity rates more directly measure contact between individuals. These points are reiterated in the discussion on Lines 297-313. The metric chosen by a manager to understand aggregation is necessarily tied to the transmission dynamics of the disease in question. We have added text to outline the implications identified here (Lines 330-332).**

Lines 288–296: The authors suggest incorporating landscape features like mineral licks as pseudo-individuals for aggregation analysis. How feasible is this with current data, especially if collar resolution or location accuracy is coarse? Can the authors elaborate on how resolution affects the ability to detect point-based aggregation patterns? This should be included in the limitations.

**We are not proposing to do this with the current dataset, per se, and collar resolution does play an important role as you point out. Line 342 has been modified to read: “Fixed points on the landscape, for example attractants like anthropogenic food sources or mineral licks, can…” In another recent manuscript, which we cite, these focal points are detectable using GPS collar data.**

**Other comments:**
-Seasonal variation in herd dynamics could be introduced in the introduction of the manuscript. Given that herd structure and aggregation patterns vary substantially between summer and winter, and this variation underpins key differences in sample size requirements and aggregation metrics, seasonal dynamics should be introduced in the Introduction. Presenting this context early would help readers better understand the ecological drivers of the study and set the stage for subsequent analyses.

**We agree that this is an important topic. We introduce seasonal variation in the introduction beginning Line 94 and more extensible Lines 132-148, and even highlight the conceptual significance of these patterns using Figure 1 (also in the introduction): “*Relative ungulate herd densities and contact probabilities fluctuate seasonally (e.g., summer versus winter) with changing biotic and abiotic conditions [33]. During winter, elk herds concentrate on relatively small winter ranges where individuals interact with more conspecifics (homogenous mixing; Fig 1a). During summer, elk disperse across the landscape which reduces relative densities, and results in fewer conspecific contacts (heterogeneous mixing; Figs 1b-c).”* We also further elaborate on the seasonal contexts related to this specific study in the Materials and Methods section Lines 185-190.**

- the seasons and differences in vegetation and landscape structure should be clearly written in the study area section in methods.

**We provide a comprehensive overview on of the biophysical contexts each herd experiences including topics such as vegetation composition, topographic landscape, elevation, and predator communities. See Lines 158-175: *“The JKSN herd inhabit the Middle Rockies ecoregion defined by steep mountains dominated by coniferous forest and shrub- and grass-covered foothills (1,500–3,000m elevation [50]). During this study, elk from the JKSN herd were observed within an 1,822 km2 area encompassing their wintering grounds on NER and summer range in the Teton and Gros Ventre Ranges, including portions of Grand Teton and Yellowstone National Parks to the west and north and Bridger Teton National Forest to the east (Fig 2). WGR elk inhabit the foothill shrublands and low conifer-dotted mountains of the Wyoming Basin ecoregion (1,500–2,200m elevation [50]). Elk from the WGR herd were observed within a 730 km2 area, including their winter range on Fossil Butte National Monument and summer range in the surrounding Tunp Range which mainly consists of Bureau of Land Management lands (Fig 2). While both herds experience predation risk from mountain lions (Puma concolor), the JKSN herd also live within the range of grizzly bears (Ursus arctos) and gray wolves (Canis lupus).”***

-Given the known differences in elk behavior and group structure between summer and winter, more explicit discussion is needed on how seasonal context influences metric sensitivity, contact rates, and implications for disease management.

**We discuss the connections highlighted by the reviewer throughout the discussion:**

**Lines 290-296: *“When elk are widely dispersed during the summer months, the overlap of space use between individuals decreases, requiring a larger sample to capture aggregation patterns for the herd. In contrast, when herds are tightly grouped, as they are on wintering grounds, an individual’s space use commonly overlaps with others in the herd. The metrics robust to small sample sizes varied both within and between herds, indicating that herd context is a crucial consideration when determining the optimum sample size and density metrics for studying aggregation behavior in big game populations.”***

-Please include a brief discussion of the study's limitations. Specifically, address potential effects of varying GPS collar resolution, the exclusive use of female elk, the fixed 500 m proximity threshold, and the absence of sensitivity analyses for sampling design. Acknowledging these will provide important context for interpreting and applying the results

**We have added text to expand on the points the reviewer has raised here. Refer to new paragraph in discussion beginning on Line 327 and other changes throughout the manuscript. We respond to each to these points specifically where they appear in the review comments above.**
