# Supplementary material for: Estimating GPS-based social aggregation metrics using collar data
Source: PLoS One. 2026 Apr 9;21(4):e0345546. doi: 10.1371/journal.pone.0345546 (PMC13065019; doi:10.1371/journal.pone.0345546)
Supplement: Attachment — Submitted filename: Response to Reviewer PONE-D-25-26403R1.docx [file pone.0345546.s003.docx]

**Response to Reviewers**

**Here we have addressed the reviewer comments found in the ‘Reviewer Questionnaire” email and the line by line responses to comments left in “Reviewer comments” PDF file we received. They are as follows:**

**Responses to email comments:**

Additional Editor Comments: Dear authors, Kindly address all the issues and concerns raised by both reviewers carefully.

Reviewers' comments:

Reviewer's Responses to Questions

**Comments to the Author**

1. If the authors have adequately addressed your comments raised in a previous round of review and you feel that this manuscript is now acceptable for publication, you may indicate that here to bypass the “Comments to the Author” section, enter your conflict of interest statement in the “Confidential to Editor” section, and submit your "Accept" recommendation.

Reviewer #1: All comments have been addressed

Reviewer #2: All comments have been addressed

**Response: We thank the reviewers for their perspectives and time.**

2. Is the manuscript technically sound, and do the data support the conclusions?

The manuscript must describe a technically sound piece of scientific research with data that supports the conclusions. Experiments must have been conducted rigorously, with appropriate controls, replication, and sample sizes. The conclusions must be drawn appropriately based on the data presented.

Reviewer #1: Yes

Reviewer #2: Partly

**Response: We have made many of the suggested changes, adding additional context and details that were requested during the first round of review and believe our submission is stronger after the peer review process.**

3. Has the statistical analysis been performed appropriately and rigorously?

Reviewer #1: Yes

Reviewer #2: I Don't Know

**Response: We believe we have exceeded the standards for statistical rigor in this study.**

4. Have the authors made all data underlying the findings in their manuscript fully available?

The [PLOS Data policy](https://gcc02.safelinks.protection.outlook.com/?url=http%3A%2F%2Ftrack.editorialmanager.com%2FCL0%2Fhttp%3A%252F%252Fwww.plosone.org%252Fstatic%252Fpolicies.action%2523sharing%2F1%2F010f019a94e1bdf4-54ac3e3e-44b1-448c-af00-5271066bee73-000000%2F_aptaYZX4JksvGC-jtv6sVFkTfliinNbOdPUXcLTTYY%3D237&data=05%7C02%7Cwjanousek%40usgs.gov%7Cb1764fb0228c435eddff08de3d8dc146%7C0693b5ba4b184d7b9341f32f400a5494%7C0%7C0%7C639015876198504690%7CUnknown%7CTWFpbGZsb3d8eyJFbXB0eU1hcGkiOnRydWUsIlYiOiIwLjAuMDAwMCIsIlAiOiJXaW4zMiIsIkFOIjoiTWFpbCIsIldUIjoyfQ%3D%3D%7C0%7C%7C%7C&sdata=s6%2BOnzyDRmfKOiNaziAwg7pVNwbwCtiQkWO%2FN3XpimA%3D&reserved=0) requires authors to make all data underlying the findings described in their manuscript fully available without restriction, with rare exception (please refer to the Data Availability Statement in the manuscript PDF file). The data should be provided as part of the manuscript or its supporting information, or deposited to a public repository. For example, in addition to summary statistics, the data points behind means, medians and variance measures should be available. If there are restrictions on publicly sharing data—e.g. participant privacy or use of data from a third party—those must be specified.

Reviewer #1: Yes

Reviewer #2: No

**Response to Reviewer #2: We have provided contact information for data not readily available due to data sensitivities surrounding information within National Parks. We have published all other underlying data in a static publicly available database (cited in text).**

5. Is the manuscript presented in an intelligible fashion and written in standard English?

PLOS ONE does not copyedit accepted manuscripts, so the language in submitted articles must be clear, correct, and unambiguous. Any typographical or grammatical errors should be corrected at revision, so please note any specific errors here.

Reviewer #1: Yes

Reviewer #2: No

**We disagree with Reviewer #2’s assessment that the paper is not written in standard English.**

6. Review Comments to the Author

Please use the space provided to explain your answers to the questions above. You may also include additional comments for the author, including concerns about dual publication, research ethics, or publication ethics. (Please upload your review as an attachment if it exceeds 20,000 characters)

Reviewer #1: Thank you for addressing all the comments and suggestion. All the reviewers' comments were anserwerd and incorporated in the revised manuscript. There are only a few minor edits that I have commented on in the manuscript file.

**Response: We appreciate the feedback we received and have addressed all minor edits in this last review round.**

Reviewer #2: After a careful review of the paper, I found that the topic pertains to ecology (environmental science), specifically the use of modern technologies to monitor wildlife, behaviors, and populations of animals in a particular area. The paper lacks the technical mechanisms of the Global Positioning System (GPS) and relies solely on data received about the situation in that area. While the research is valuable in environmental science and related fields, and does contribute to those areas, its contribution to the technology itself is insufficient for publication.

GPS data is available for use by researchers in numerous scientific fields, and here the researchers have used this data to monitor and study the behavior of a group of wild animals.

**Response: The reviewer critiques the paper for not advancing GPS technology, stating that its contribution to technology is insufficient for publication. However, nowhere in the manuscript do we claim to be advancing GPS technology. The study clearly focuses on the ecological application of GPS collar data to investigate aggregation behavior in elk, which we believe is well within the scope of the journal.**

**Line by line responses:**

**Line 17.** Added the word ‘As’.

**Line 18.** Deleted the word ‘and’ and added comma per reviewer’s request.

**Line 146:** Reviewer comment: “*I suppose Figure 2 will come here in text.*” **We imagine this will be an editorial decision on the journal’s part in terms of formatting but yes this paragraph is where Figure 2 is introduced.**

**Line 151:** Reviewer comment: “*Consufed about this figure numbers. Kindly please check again.*” **We have double-checked the figure numbering to ensure they are correct and streamlined where the figures are mentioned in the paragraph in question so that they appear in logical order.**
